# Disentangling on and off-target binding in flortaucipir PET: a voxel-to-voxel P-tau, ferric iron, and MAO-B histology-to-flortaucipir PET comparison

**DOI:** 10.1007/s00401-026-02983-x

**Published:** 2026-02-25

**Authors:** Yuheng Chen, Renaud La Joie, Felipe L. Pereira, Ganna Blazhenets, Lucile Zhu, Salvatore Spina, William W. Seeley, Helmut Heinsen, Daniela Ushizima, Duygu Tosun, Gil D. Rabinovici, Lea T. Grinberg

**Affiliations:** 1 Memory and Aging Center, Weill Institute for Neurosciences, University of California San Francisco, San Francisco, CA 94158 USA; 2 Departments of Laboratory Medicine and Pathology and Neuroscience, Mayo Clinic Florida, 4500 San Pablo Road South, Jacksonville, FL 32224 USA; 3 Department of Quantitative Health Science, Mayo Clinic, Jacksonville, FL 32224 USA; 4 Institute of Forensic Medicine, Julius-Maximilians-Universität, 97078 Würzburg, Germany; 5 Center for Advanced Mathematics for Energy Research Applications, Lawrence Berkeley National Laboratory, Berkeley, CA 94720 USA; 6 Computational Research Division, Lawrence Berkeley National Laboratory, Berkeley, CA 94720 USA; 7 Department of Radiology and Biomedical Imaging, University of California San Francisco, San Francisco, CA 94107 USA

**Keywords:** Flortaucipir PET, Tau pathology, Off-target binding, Monoamine oxidase B (MAO-B), Iron deposits, Voxel-to-voxel comparisons

## Abstract

Flortaucipir PET imaging has significantly advanced our ability to visualize tau pathology in vivo. However, off-target Flortaucipir signal remains a considerable challenge for interpreting of imaging results, particularly in non-Alzheimer's tauopathies and non-tau pathologies. To better understand this off-target signal, we used an innovative voxel-to-voxel correlation approach, analyzing thousands of histology-Flortaucipir pairs from individual cases. This allowed us to quantitatively assess the relationship between Flortaucipir PET signal and three key biological factors: histological tau burden (CP-13 phospho-tau), ferric iron (Perls’ Prussian blue), and monoamine oxidase B (MAO-B). Our study included individuals with Alzheimer's disease (AD), various non-AD tauopathies, and a case of FTLD-TDP-43 type A. In AD, Flortaucipir signal showed a significant but moderate correlation with histological tau pathology, suggesting that while tau is a major contributor, other biological factors also influence Flortaucipir binding in AD. Conversely, in non-AD tauopathies and FTLD-TDP-43, correlations between Flortaucipir signal and tau pathology were weak or negligible. Instead, Flortaucipir signal correlated more strongly with ferric iron and MAO-B. However, these factors did not fully explain all the off-target signals, implying other unknown contributors are likely involved. These findings underscore the complexity of interpreting Flortaucipir PET scans. A thorough understanding of off-target binding mechanisms is crucial for improving the diagnostic accuracy of Flortaucipir PET and its specificity.

**Supplementary Information:**

The online version contains supplementary material available at 10.1007/s00401-026-02983-x.

## Introduction

Neurodegenerative disorders are the leading cause of dementia in the aging population, affecting over 6 million individuals in the United States alone. These disorders are characterized by the buildup of abnormal proteins, which leads to neuronal dysfunction and death. One of the most significant proteins associated with neurodegenerative disorders is the misfolded tau protein, a pathological hallmark of Alzheimer's disease (AD) and over 20 other sporadic and inherited neurodegenerative disorders collectively known as tauopathies [10]. Until recently, postmortem examinations were the only means of detecting abnormal tau deposits in the brain.

The development of tau positron emission tomography (PET) tracers has enabled the in vivo detection and mapping of tau pathology. [18F] flortaucipir PET (Flortaucipir), formerly known as AV-1451, the first FDA-approved tau PET tracer, is widely used in research and clinical trials [8]. Numerous studies have demonstrated that Flortaucipir can effectively detect AD-related tau, particularly in moderate to severe stages of the disease [16, 27, 34]. However, Flortaucipir exhibits significantly lower retention in non-Alzheimer's tauopathies, such as progressive supranuclear palsy (PSP), corticobasal degeneration (CBD) and chronic traumatic encephalopathy [9, 18, 19, 32].

A major clinical challenge with Flortaucipir PET is the frequent detection of signals in regions not expected to harbor significant tau pathology in AD [1, 14]. Off-target retention is particularly prominent in the basal ganglia, substantia nigra, red nucleus, and choroid plexus [26]., where tracer kinetics and regional signal patterns are inconsistent with neurofibrillary tangle distribution. In addition, studies have documented elevated Flortaucipir uptake in cortical regions in semantic variant primary progressive aphasia and in FTLD due to progranulin mutations, disorders that are almost invariably associated with TDP-43 rather than tau pathology [1, 17, 35, 38].

Dysregulation of ferric iron—controlled by ferritin, the primary protein for iron storage and release—is closely linked to AD pathology [13], while imaging studies show associations between ferric iron accumulation and non-AD tauopathies [29]. Age-related ferric iron increases are particularly evident in the putamen, caudate, substantia nigra, and red nucleus [28], overlapping with regions showing off-target Flortaucipir retention, where Flortaucipir frequently colocalizes with ferric iron accumulation [2, 4]. These observations suggest that part of the Flortaucipir signal in PSP and CBD may reflect binding to ferric iron or related molecules [12, 35] rather than tau itself, consistent with reports showing weak correlations between Flortaucipir uptake and 4-repeat tau pathology [4, 9, 35]. Supporting this interpretation, autoradiography and imaging studies have identified ferric iron deposits as potential substrates for non-tau-related Flortaucipir binding [11, 15, 21].

Monoamine oxidase B (MAO-B), a known source of off-target signal for other first-generation tau PET tracers such as [18F]-THK5351 [2, 24, 40], has also been proposed as a potential contributor to off-target Flortaucipir signal (Baker, Harrison et al.). MAO-B is enriched in astrocytes and is upregulated in reactive astrogliosis [7], which is common in neurodegenerative disease and may spatially overlap with regions showing elevated Flortaucipir retention. Evidence for MAO-B as a driver of Flortaucipir signal, however, remains conflicting. In vitro, Flortaucipir has been reported to bind monoamine oxidases, including MAO-B [40]. In vivo, by contrast, MAO-B inhibitors do not block Flortaucipir binding [6], and modeling work suggests MAO-related binding is unlikely to meaningfully impact typical clinical interpretation of Flortaucipir PET [41]. Together, these mixed findings make it difficult to determine whether Flortaucipir retention in disorders with prominent astrogliosis reflects tau, MAO-B–related off-target binding, or a combination of substrates [2]. This uncertainty highlights the need for direct, spatially resolved testing to disentangle the relative contributions of tau and non-tau substrates.

While several studies have investigated the biological basis of Flortaucipir by comparing PET results with histological data, methods that allow for point-to-point correlations between neuroimaging voxels and corresponding histological counterparts, which are ideal for elucidating the neurobiological basis of imaging signal, have rarely been applied to studies focusing on Flortaucipir on-target and off-target signal. To address this, we utilized a novel, convolutional neural network (CNN) based, *in-house* method that enables voxel-to-voxel correlation of quantitative histological data with spatially matched PET signal values from the same individuals [39]. We aimed to investigate the relative contributions of phospho-tau, ferric iron, and MAO-B to the Flortaucipir signal in AD, other tauopathies, and a case of frontotemporal lobar degeneration (FTLD) with transactive response DNA binding protein 43 kDa (TDP‑43) type A due to a progranulin mutation. Understanding the neurobiological basis of the Flortaucipir signal will enhance PET signal interpretation, thereby increasing the value of this tracer in diagnosing neurodegenerative disorders, assessing neuropathological burden, monitoring disease progression, and evaluating the effectiveness of tau-targeting therapies.

## Materials and methods

### Case selection

This study was approved by the University of California, San Francisco (UCSF) institutional review board. Post-mortem human brains were obtained from the Neurodegenerative Disease Brain Bank (NDBB) of UCSF Memory and Aging Center, which performs autopsies in participants of the AD Research Center (ADRC) and other studies. This study included five subjects who underwent in vivo magnetic resonance imaging (MRI) and Flortaucipir PET. The PET-to-autopsy interval was between 0.75 to 4.16 years. Upon death, each brain received a standardized neuropathological assessment for neurodegenerative diseases that followed universally accepted guidelines. AD neuropathological changes (ADNC) were scored according to the latest guidelines [23]. Inclusion criteria included a post-mortem interval of under 24 h, no more than one primary neuropathological diagnosis, lack of Axis I psychiatric disorder diagnosis, or non-dementia neurological disorder, or gross non-degenerative structural neuropathology. To provide a broad representation of tauopathies, we included these cases: AD (3R/4R tauopathy: both pathological 3-repeated and 4-repeated tau isoforms), CBD (4R tauopathy: majority of pathological 4-repeated tau isoforms), FTLD with MAPT mutation S305I (FTLD-MAPT) (4R tauopathy), PSP (4R tauopathy), as well as a non-tauopathy case, an FTLD-TDP type A (FTLD-TDP), due to a progranulin mutation (Table 1).

**Table 1:** Demographic, Flortaucipir PET, and diagnosis information

Case	AD	PSP	CBD	FTLD-MAPT-S305I	FTLD-TDP
Age at PET (yrs)	73	60	63	38	68
Clinical diagnosis	lvPPA	PSP-RS	PSP-RS	bvFTD	bvFTD
Primary neuropathology diagnosis	AD	FTLD-Tau (4R), PSP	FTLD-Tau (4R), CBD	FTLD-Tau (4R), MAPT-S305I	FTLD-TDP-43 type A
Braak stage	VI	III	I	0	I
Secondary neuropathological diagnosis	Limbic-transitional LBD	Intermediate ADNC	none	none	none
Tertiary neuropathological diagnosis	ARTAG	AGD			
ABC score	A3B3C3	A3B2C1	A0B1C0	A0B0C0	A1B1C0
Time between flortaucipir PET and autopsy (yrs)	3.14	4.16	0.75	2.14	1.5
Flortaucipir PET location	Lawrence Berkeley Lab	Lawrence Berkeley Lab	Lawrence Berkeley Lab	Lawrence Berkeley Lab	UCSF China Basin
Flortaucipir PET machine	Siemens Biograph PET/CT	Siemens Biograph PET/CT	Siemens Biograph PET/CT	Siemens Biograph PET/CT	GE Discovery VCT SV PET/CT

yrs, years; lvPPA, logopenic variant primary progressive aphasia; PSP-RS, progressive supranuclear palsy—Richardson's syndrome; bvFTD, behavioral variant frontotemporal dementia; AD, Alzheimer’s disease; FTLD-Tau, frontotemporal lobar degeneration with tau pathology; CBD, corticobasal degeneration; FTLD-TDP-43, frontotemporal lobar degeneration with TDP-43, type A pathology

### PET and MRI acquisition and preprocessing

MRI sequences were acquired at UCSF, either on a 3 T Siemens Tim Trio (n = 3) or a 3 T Siemens Prisma Fit (n = 2) scanner. For PET processing, we used T1-weighted magnetization prepared rapid gradient echo (MPRAGE) images acquired using standard protocols (sagittal slice orientation; 1 × 1 × 1 mm resolution; 160 slices per slab; 240 × 256 matrix; repetition time = 2.3 ms; inversion time = 900 ms; flip angle = 9°; echo time = 2.98 ms for Trio, 1.9 ms for Prisma). MRIs were segmented and parcellated using FreeSurfer 5.3 (DownloadAndInstall5.3—Free Surfer Wiki). Statistical Parametric Mapping (SPM12; Wellcome Trust Center for Neuroimaging, London, UK, https://www.fil.ion.ucl.ac.uk/spm) was used to process PET data as described below.

Flortaucipir PET images were acquired on a Siemens Biograph PET/CT scanner at Lawrence Berkeley National Lab (n = 4) or a GE Discovery STE/VCT PET/CT scanner at the UCSF Department of Radiology and Biomedical Imaging at China Basin (n = 1). We analyzed PET data acquired 80–100 min (four 5-min frames) after injection of 10 mCi of Flortaucipir. A low-dose CT scan was performed for attenuation correction, and data were reconstructed using an ordered subset expectation maximization algorithm with weighted attenuation and scatter correction without smoothing. PET frames were realigned, averaged, and coregistered onto their corresponding MRI. Standardized uptake value ratio (SUVR) images were created in native space using MRI-defined inferior cerebellum gray matter as a reference region [36]. Quality control for Flortaucipir PET signal registrations and SUVR thresholding was performed by R.L., including evaluation using Dice coefficients.

### Project-specific neuropathological processing and staining

Figure S1 summarizes the methodological workflow, from imaging acquisition to the final PET-to-histology registered datasets used for analysis.

Upon autopsy at the NDBB, the brains were coronally sectioned into approximately 1 cm slabs. Half of these slabs were immediately frozen, while the remaining were immersion-fixed in 4% PFA for 72 h. Subsequently, fixed slabs were transferred to phosphate-buffered saline (PBS) with azide and stored at 4 °C. A subset of the fixed slabs was sampled for neuropathological diagnosis. For this study, we selected brain slabs based on these criteria: (1) slabs not used for neuropathological diagnosis; (2) out of those, slabs covering brain areas showing Flortaucipir signal; (3) whenever possible, slabs containing the basal ganglia (a well-known source of off-target Flortaucipir signal). Using the criteria described above, the imaging team selected the most appropriate slide for each case, and the neuropathology team remained blinded to PET scan results until completion of histological analysis and histology–MRI registration. In total, we processed and investigated six coronal hemispheric slabs: two from an AD case (*i.e.*, specifically at the level of the corpus callosum (CC) and posterior hippocampus (PH)), and one each from these cases: at the level of anterior hippocampus (PSP and FTLD-TDP), at the level of lateral geniculate body (CBD), and amygdala (FTLD-MAPT-S305I). The selected slabs were embedded in celloidin using an established protocol and then cut into serial 160 µm-thick coronal sections. Brain cutting was done using a sliding microscope equipped with a photo stand and camera. This setup allowed for the acquisition of blockface images (photographs of the slab surface after each cut), which are essential for subsequent 2D/3D image registration and reconstruction. The brain processing methods are detailed in our previous study [39]. From the resulting serial coronal sections (around 20 per slab), the adjacent full-face sections were selected for histological staining. The first section was immunostained for p-tau (1:400, CP-13 [p-tau Ser 202,] mouse, gift from Peter Davis, developed with Impact DAB). The tau antibody choice was based on a pilot study comparing how well three different tau antibodies (CP-13, PHF-1 [ptau Ser 396/404]) and MC-1 [that capture conformational changes], data not shown, and the parallel sections underwent immunostaining for MAO-B (1:1800, HPA002328, Rabbit, Sigma Aldrich, developed with Impact DAB) or ferric iron staining (Perls’ Prussian blue). Perls’ Prussian blue was used to localize ferric iron (Fe^3^⁺) sequestered in ferritin/hemosiderin, providing specificity for non-heme storage iron [37]. Representative blockface images and corresponding low- and high-magnification CP-13, ferric iron (Perls’), and MAO-B staining across all cases are shown in Fig. S2.

### Digitizing histological images, segmenting histological signals, and quantitative histological heatmaps

We used our *in-house*-*built* scanner, designed for large slices, to scan the mounted slides at a spatial resolution of 1.22 µm/pixel [39] (Fig S3). Each slide was scanned at three different depths, with an interval of ~ 53 µm between scans. To enhance resolution and contrast without altering color information, we applied a focus stack using the Enfuse library (https://enblend.sourceforge.net/) [22]. Each slide was digitally captured as 500 to 1500 image tiles, each approximately 8.8 mm^2^. These tiles were then stitched to reconstruct a full-coronal view of the slide [39]. An example of the full workflow from scanned images to probability maps and thresholded quantitative segmentation maps is shown in Fig. S4. The resulting digital images were processed using our IHCNet segmentation pipeline (Fig. S5). This UNet (one of the CNN architectures) based tool is specifically designed for challenging segmentation tasks in antibody-stained images, effectively separating foreground signal from complex backgrounds and protein structure, such as those found in p-tau/CP-13 and MAO-B staining [39]. For segmenting the p-tau/CP-13 signal, we expanded our existing IHCNet model-training dataset by including images from various tauopathies, as well as MAO-B staining images. The p-tau training dataset comprises 254 (1024 × 1024 pixels) randomly selected, expert-labeled p-tau images, then augmented with stride-tiling (stride-step-size is 90 pixels), resulting in a total of 36,576 training dataset images. In a similar pipeline, the MAO-B training dataset contained 38,448 labeled images. Both datasets are then trained with IHCNet. To maximize segmentation sensitivity and accuracy, the optimal thresholds for each marker were determined using Precision-Recall, and Receiver Operating Characteristic (ROC) curves are used to evaluate model performance (Fig. S6). Precision and Recall calculations are formulated as follows:

$$\begin{aligned} {\mathrm{Precision}} & = \frac{{{\mathrm{correctly}}\,{\mathrm{classificed}}\,{\mathrm{actual}}\,{\mathrm{positives}}}}{{{\mathrm{everything}}\,{\mathrm{classified}}\,{\mathrm{as}}\,{\mathrm{positive}}}} = \frac{True\,Positive}{{True\,Positive + False\,Positive}} \\ {\mathrm{Recall}} & = \frac{{{\mathrm{correctly}}\,{\mathrm{classificed}}\,{\mathrm{actual}}\,{\mathrm{positives}}}}{{{\mathrm{all}}\,{\mathrm{actual}}\,{\mathrm{positive}}}} = \frac{True\,Positive}{{True\,Positive + False\,Negative}} \\ \end{aligned}$$

where Precision indicates the proportion of model’s positive classification that are positive, and Recall indicates the proportion of all actual positive that were classified accurately. ROC curve was graphed with true positive rate over false positive rate at different threshold levels. Higher area-under-curve (AUC, range from 0 to 1) indicates more robust model prediction to true positive.

Due to the less complicated background and consistent color of Perls’ blue staining for ferric iron, we used the Trainable-Weka-Segmentation tool in Fiji for ferric iron signal segmentation (Fig S7); its classifiers were sufficient for this color-based staining. While brightfield immunohistochemistry is primarily qualitative due to the challenges in normalizing DAB signal intensity, our segmentation process provides quantitative topographical maps that indicate the percentage of each voxel occupied by the signal of interest. Histological staining quality was independently assessed by a senior neuropathologist.

The computational pipeline used to generate quantitative histological signal heatmaps has been detailed in a previous methodology paper by our group and is accompanied by publicly available source code. In brief, the segmentation outputs from CNN-based models (generated by IHCNet for p-tau/CP-13 and MAO-B) and Fiji/ImageJ Weka segmentation (for ferric iron) yield probability maps. For CP-13 and MAO-B, IHCNet outputs a probability between 0 and 1 for each pixel. Using precision–recall curve analysis and expert visual review, we selected a single global probability threshold of 0.7; pixels with probability ≥ 0.7 were labeled as positive and < 0.7 as background, yielding the binary maps used for downstream quantification (Fig. S6). Subsequently, these binary maps are used to calculate the percentage of positive pixels within spatial areas equivalent to 1 mm^2^, thereby approximating MRI resolution. For ferric iron, the Trainable Weka Segmentation model similarly outputs per-pixel probabilities, which were binarized at a threshold of 0.93 chosen from the precision–recall curve based on the F1 score (Fig. S7). These values are then reconstructed into downsampled heatmaps at MRI resolution (also detailed in the Methods section). The heatmaps are further processed using 3DFWHMx (AFNI, NIH) to estimate the full width at half-maximum (FWHM) for each Flortaucipir PET dataset. FWHM matching is then performed using 3DBlurToFWHM (AFNI, NIH) to align heatmap resolution with Flortaucipir data, ensuring accurate correlation analyses.

### PET to histology registration and choice of ROIs for analysis

To enable voxel-to-voxel correlation between histology and PET signal, we implemented a multi-step registration and data processing pipeline: (1) 2D histological registration: In order to correct the tissue distortion/tearing due to immunohistochemistry processing, the stitched images of stained histological slides were registered to their corresponding blockface image with affine registration using thin-plate spline with landmark in MPIAV, follow with Symmetric Normalization (SyN) registration. (2) Quantitative map application: This registration transformation was then applied to the quantitative heatmaps obtained from segmenting p-tau/CP-13, ferric iron, and MAO-B signal. (3) 3D histological reconstruction: The blockface images were subsequently stacked to generate a 3D histological volume [39]. The resulting 3D reconstructions and the MRI/PET-aligned blockface/histology/heatmaps used for statistical analysis are illustrated in Fig. S8. Next, the 3D blockface volume was linearly registered to the MRI using FreeSurfer’s Freeview tool, with visual inspection to ensure accurate alignment. Subsequently, the corresponding MRI, PET scan, and the registered blockface images were imported into MIPAV for the second round of thin-plate spline registration, following with SyN registration to refine alignment across modalities. These steps allow resampling of the high-resolution blockface and quantitative heatmap data to the lower resolution space of the MRI and PET images, ensuring precise anatomical overlap and maximizing registration accuracy. To facilitate voxel-to-voxel correlation analysis across different datasets, it was necessary to standardize their spatial resolution. The high-resolution histological images (originally 1.22 µm/pixel) were smoothed using a Gaussian filter to match MRI intrinsic resolution (1 mm isotropic), and subsequently to PET resolution (5 mm isotropic). While necessary for integrating data across scales, this resolution adjustment may underestimate the true heterogeneity present in the original high-resolution histological images, as down-sampling can blur fine structural details and diminish the apparent intensity of focal histological signal.

Our main goal was to investigate the contribution of p-tau/CP-13, ferric iron, and MAO-B histological burden to the Flortaucipir signal. Thus, the Flortaucipir signal was designated as the outcome variable in our regression models. Since in most cases the Flortaucipir signal was restricted to a few brain areas, we created ROIs based on Flortaucipir maps rather than attempting whole-brain correlations with histological maps. To generate these ROIs, a binary threshold was applied to the PET images, including all voxels with SUVR values greater than 1.2. This cutoff is commonly used to exclude background noise and to define Flortaucipir positivity [25]. SUVR values above the threshold were analyzed as a continuous variable. The threshold was selected based on sensitivity analyses using alternative SUVR cutoffs (1.2, 1.3, and 1.4; Table S1). The overall pattern of results and the main conclusions remained unchanged across thresholds, supporting the use of an SUVR threshold of 1.2. Each histological slide contains multiple such ROIs. These ROIs were then applied to the corresponding registered neuropathological staining maps (*i.e.*, p-tau/CP-13, ferric iron, MAO-B). Subregional ROIs for the basal ganglia (*i.e.*, putamen and globus pallidus, collectively referred to as the lentiform nucleus) were delineated based on anatomical boundaries defined in both MRI and histological spaces. Correlation and regression analyses were performed separately for each structure, as well as in combination, to investigate regional differences in the sources contributing to the Flortaucipir PET signal.

### Statistical analysis

Spearman correlation tests were performed between each histological marker and Flortaucipir values using the cor.test function in R, with results visualized using the ggplot2 package. Multiple linear regression models were applied to evaluate the contribution of each predictor to the Flortaucipir outcome. Both Spearman correlation and mixed linear models are applied to the same dataset to capture complementary aspects of the data: Spearman assesses monotonic associations, while mixed models account for covariates and intra-group variability, enabling a more robust evaluation of the underlying relationships. For tauopathy cases (*i.e.*, AD, PSP, CBD, and FTLD-MAPT-S305I), these models were tested: *m*0 = Flortaucipir ~ p-tau/CP-13 + ferric iron + MAO-B; *m*1 = Flortaucipir ~ p-tau/CP-13 + ferric iron; *m*2 = Flortaucipir ~ p-tau/CP-13 + MAO-B; *m*3 = Flortaucipir ~ ferric iron + MAO-B; *m*4 = Flortaucipir ~ p-tau/CP-13; *m*5 = Flortaucipir ~ ferric iron; and *m*6 = Flortaucipir ~ MAO-B. These models were designed to isolate and test the unique contribution of each predictor (p-tau/CP-13, ferric iron, and MAO-B) to Flortaucipir binding by systematically varying model composition. For the non-tauopathy case (*i.e.*, FTLD-TDP), only models *m*3, *m*5, and *m*6 were used. Models *m*4-*m*6 differ from correlation analyses by accounting for directionality. Model comparison was conducted using the Akaike Information Criterion (AIC), calculated with the aictab function from the aiccmodavg package. All reported ΔAIC values indicate the relative model quality compared to *m*0. Collinearity between predictors was assessed using variance inflation factors (VIF), all of which were below 2, indicating that multicollinearity was not a significant concern. The Dice coefficient was used to quantify registration accuracy between histological and in vivo images (MRI/PET) by comparing binary masks of the registered/transformed blockface (histological) images with the corresponding MRI/PET images (Fig. S9). A high Dice score, approximately ≥ 0.8, indicates good registration accuracy. In addition to the formal correlation and regression models, we generated descriptive voxel-wise scatter/hexbin plots of Flortaucipir SUVR versus each histological marker (CP-13, ferric iron, and MAO-B) for each case. These plots were stratified by major regions of interest (full hemisphere, cortical areas, white matter, lentiform nucleus, and hippocampus, when applicable). The hexbin color scale represents the number of voxels within each combination of histological burden and SUVR. These plots were used to visually compare the distribution of Flortaucipir signal at similar levels of ferric iron or MAO-B across regions and diagnoses (Fig. S10). All analyses were conducted using R software (version 4.2.2; R Foundation for Statistical Computing, Vienna, Austria).

## Results

### Flortaucipir PET and performance of CNN-based histological segmentation

We evaluated the CNN models used for segmenting p-tau/CP-13 and MAO-B histological markers by analyzing Precision-Recall and ROC curves (Fig. S6). Both models achieved ROC AUC values ≥ 0.8, indicating good accuracy across various staining backgrounds and signal intensities. Based on Precision-Recall curve analysis and expert review by a senior neuropathologist blinded to diagnosis, we selected a threshold of 0.7 for converting probability maps into binary segmentations suitable for downstream quantification (Fig. S6).

To assess the accuracy of registering histological sections to MRI scans, Dice coefficients were calculated (Fig. S9). Across entire slabs, Dice scores ranged from 0.889 to 0.949, reflecting excellent alignment. In the lentiform nucleus, scores ranged from 0.76 to 0.90, which we deemed acceptable given its structural complexity and tissue processing distortions.

### Cohort and imaging overview

**Fig. 1:**
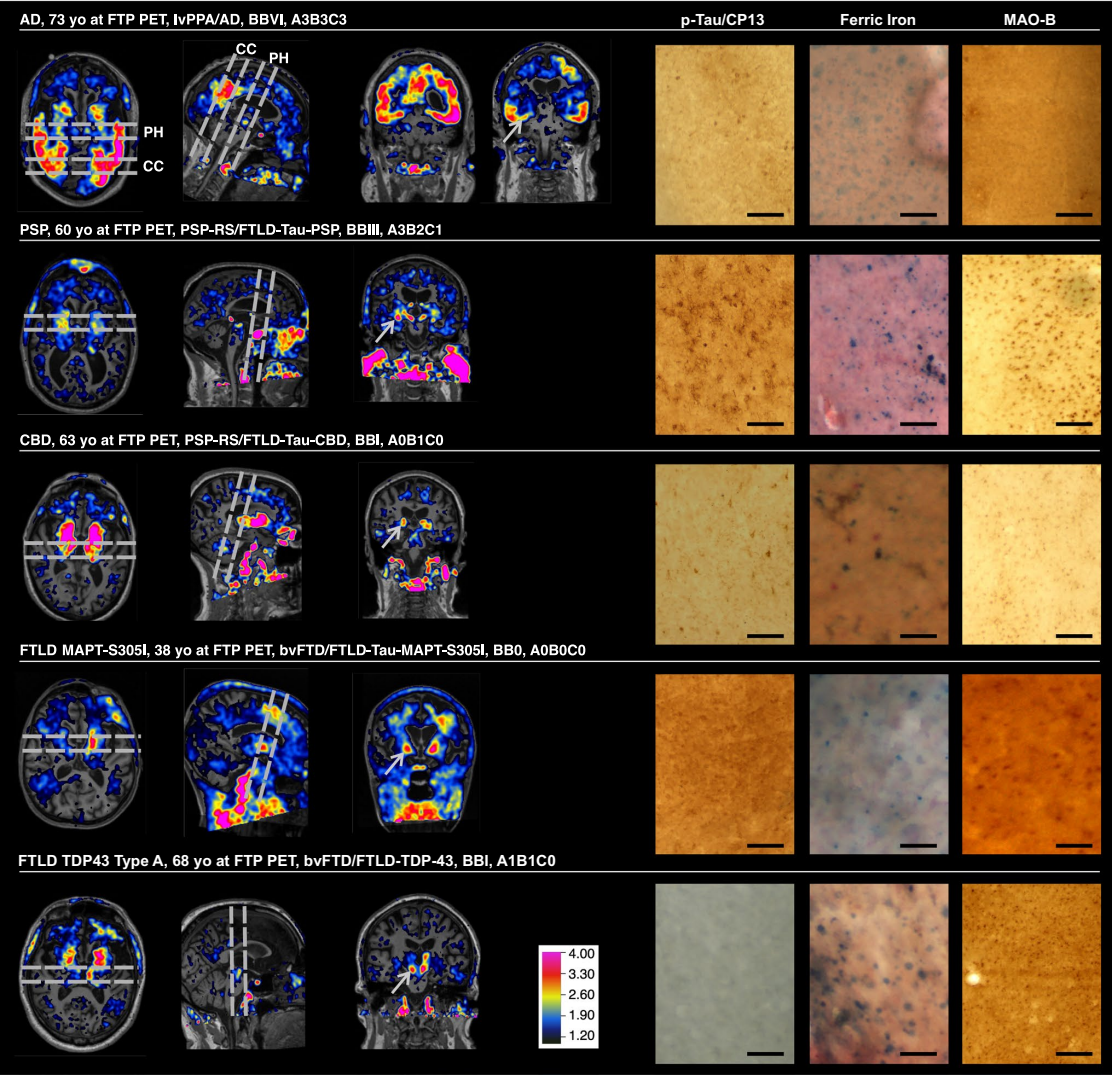
Anatomical tissue-sampling locations and corresponding histology for each case. Anatomical tissue-sampling locations are shown for each of the six histological slabs. Three MRI cross-sectional views are displayed for each case (AD, PSP, CBD, FTLD-MAPT-S305I, and FTLD-TDP), with corresponding flortaucipir PET images co-registered to the MRI scans. Gray dashed lines on the temporal and sagittal views indicate the plane of tissue sectioning, which was performed parallel to the coronal plane. White arrows on the MRI/flortaucipir PET coronal plane mark the anatomical locations of the histological samples shown on the right. For the AD case, two brain slabs were sampled (corpus callosum [CC] and posterior hippocampus [PH]). For the remaining cases, a single slab was sampled from the following regions: anterior hippocampus (PSP and FTLD-TDP), lateral geniculate body (CBD), and amygdala (FTLD-MAPT-S305I). The columns on the right depict histological representations of each case, (immuno)stained for CP-13 (p-tau), ferric iron, and MAO-B. Scale bars represent 145 μm. Histological images appear slightly blurred because the histological slides were 160 μm thick, compared with the 5–8 μm sections typically used in diagnostic neuropathology. Abbreviations: AD, Alzheimer’s disease; PSP, progressive supranuclear palsy; CBD, corticobasal degeneration; FTLD, frontotemporal lobar degeneration; MAPT, microtubule-associated protein tau; TDP, TAR DNA-binding protein 43; CC, corpus callosum; PH, posterior hippocampus; MRI, magnetic resonance imaging; PET, positron emission tomography; FTP, flortaucipir; CP-13, phospho-tau (Ser202) antibody; MAO-B, monoamine oxidase B

Figure 1 displays the co-registered Flortaucipir PET on MRI alongside corresponding p-tau/CP13, ferric iron, and MAO-B staining results for each of the 6 histological slabs.

Hemisphere ROI masks generated via FreeSurfer had volumes of approximately 3800–4200 mm^3^ per case. Lentiform nucleus masks ranged from 200 to 300 mm^3^. After applying a 1.2 SUVR threshold (to exclude background noise), retained voxel volume within hemisphere masks ranged from 2400 to 3100 mm^3^. For the lentiform nucleus, the voxel count remained close to the mask size, consistent with high Flortaucipir PET signal in this region. Only the masked and thresholded Flortaucipir PET and histological data were included in the statistical analyses.

### Alzheimer’s disease case

**Fig. 2:**
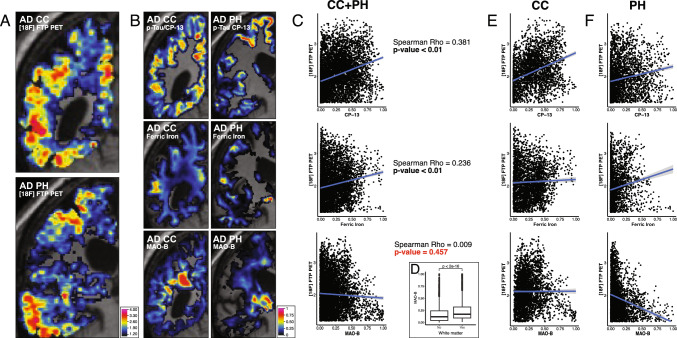
Coronal MRI views of the Corpus Callosum and Posterior Hippocampus, and their correlation with CP-13, Ferric iron, and MAO-B markers from histological staining data. **A** Flortaucipir PET SUVR maps (1.2 [blue] to 4.0 [red]; top) and the MRI hemisphere masks used as regions of interest (ROIs; bottom) for the corpus callosum (CC; left) and posterior hippocampus (PH; right). **B** Quantitative histological signal (scaled 0–1) mapped to the same MRI space as the flortaucipir PET: CP-13 immunostaining for p-tau (Ser202; top), Perls’ Prussian blue staining for ferric iron (middle), and immunostaining for MAO-B (bottom). **C** Scatter plots showing voxel-wise flortaucipir PET SUVR (y-axis) versus CP-13 (top; x-axis), ferric iron (middle; x-axis), and MAO-B (bottom; x-axis) intensities across both slabs. Blue lines indicate linear fits (flortaucipir ~ marker), and Spearman’s ρ and *p* values are shown for each marker. **D** Distribution of MAO-B voxel intensities inside versus outside white matter; Wilcoxon rank-sum *p* value is reported. **E**, **F** Scatter plots analogous to panel **C**, stratified by slab, for the CC (**E**) and PH (**F**) ROIs. Abbreviations: AD, Alzheimer’s disease; CC, corpus callosum; CP-13, phospho-tau (Ser202) antibody; FTP, flortaucipir; MAO-B, monoamine oxidase B; MRI, magnetic resonance imaging; PET, positron emission tomography; PH, posterior hippocampus; ROI, region of interest; SUVR, standardized uptake value ratio; WM, white matter

Combining data from the PH and CC slabs (Fig. 2A, B), Flortaucipir PET signal correlated significantly with p-tau/CP-13 burden (Spearman’s ρ = 0.38, *p* < 2e−16) and weakly with ferric iron (ρ = 0.23, *p* < 2e−16) but not with MAO-B (ρ < 0.01, *p* = 0.46) (Fig. 2C, Table 2).

**Table 2:** Spearman correlation and multilinear regression model metrics

	AD	AD/CC	AD/PH	PSP	CBD	FTLD-MAPT-S305I	FTLD-TDP
Flortaucipir PET spearman correlation (rho)							
p-tau/CP-13	0.38**	0.41**	0.27**	0.06**	− 0.02	− 0.10**	
Ferric iron	0.23**	0.02	0.24**	0.008	0.15**	0.22**	0.33**
MAO-B	0.01	0.10**	− 0.21**	0.17**	0.07*	0.46**	0.41**
Applied multilinear model	***m*0**	***m*0**	***m*0**	***m*0**	***m*0**	***m*0**	***m*3**
R-squared	0.131	0.152	0.101	0.044	0.026	0.226	0.245
Beta coefficients							
p-tau/CP-13	0.73**	0.89***	0.39**	0.03^ns^	− 0.05^ns^	− 0.18**	
Ferric iron	0.38**	0.27***	0.61**	0.11*	0.19**	0.10*	0.08^ns^
MAO-B	− 0.24**	− 0.10*	− 0.68**	0.24**	0.04*	0.50**	0.24**
ΔAIC *m*1	48	**1.97**	140	47	2.91	433	

AD, Alzheimer’s disease case composition of both studies regions; AD/CC, Alzheimer’s disease case corpus callosum; AD/PH, Alzheimer’s disease case posterior hippocampus; PSP, PSP case; CBD, CBD case; FTLD-MAPT-S305I, FTLD-MAPT-S305I case; FTLD-TDP, FTLD-TDP-43 type A case; ns, not statistically significant

*p* values: * < 0.05, ** < 0.01, *** < 0.001

Multilinear regression (model m0) showed p-tau/CP-13 had a ~ 1.5-fold greater contribution to Flortaucipir PET signal (β = 0.73, *p* < 2e−16) than ferric iron (β = 0.38, *p* < 2e−16). MAO-B contribution was inversely associated (β = − 0.24, *p* < 1.2e−12), likely due to high MAO-B signal in white matter regions without corresponding Flortaucipir retention (Fig. 2D). The stratified voxel-wise plots (Fig. S10) provide additional context regarding regional differences at comparable non-tau histological burdens.

Slab-specific analyses revealed:

- In CC (Fig. 2E), Flortaucipir PET strongly correlated with p-tau/CP-13 (ρ = 0.41, *p* < 2e−16), but not ferric iron (ρ = 0.02, *p* = 0.19). MAO-B correlated weakly (ρ = 0.10, *p* < 2e−16), but multilinear models (*m*0 and *m*1) showed similar AIC values, suggesting that p-tau/CP-13 and ferric ironburdens alone can explain the Flortaucipir signal as effectively as when accounting for MAO-B burden. P-tau contributed ~ 3.3 times more than ferric iron (β_p-tau = 0.89 vs. β_ferric iron = 0.27) to Flortaucipir signal.
- In PH, correlations between Flortaucipir PET and both p-tau/CP-13 (ρ = 0.27) and ferric iron (ρ = 0.24) were weak but significant (both *p* < 2e−16), while MAO-B showed a negative correlation (ρ = − 0.21) (Fig. 2F). The multilinear *m*1 suggested that ferric iron inclusions contributed more than p-tau to Flortaucipir signal variability (β_ferric iron = 0.61 vs. β_p-tau = 0.39, R2 = 0.101). Notably, the PH slab includes the lentiform nucleus, which accumulates ferric iron with age and is a known source of Flortaucipir off-target binding. The analysis of isolated lentiform nucleus is presented below.

### PSP, CBD, and FTLD-MAPT-S305I: 4R tauopathies

**Fig. 3:**
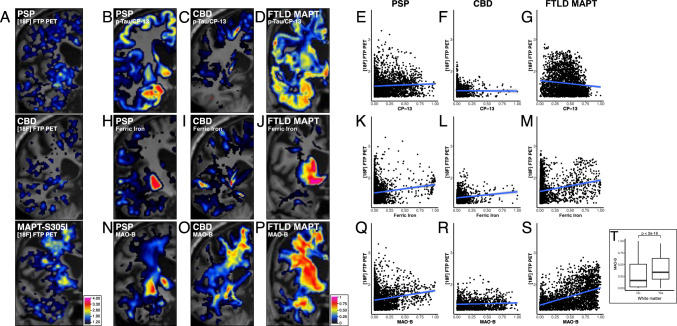
Coronal MRI views of the non-AD tauopathies, Flortaucipir PET data, and their correlation with CP-13, Ferric iron, and MAO-B markers from histological staining data. **A** Flortaucipir PET SUVR maps (1.2 [blue] to 4.0 [red]; top) and MRI hemisphere ROIs (bottom) for the PSP case (anterior hippocampus, left), CBD case (lateral geniculate body, middle), and FTLD-MAPT-S305I case (amygdala, right). **B**–**D**, **H**–**J**, **N**–**P** Quantitative histological signal (scaled 0–1) in the same MRI space as the flortaucipir PET: CP-13 immunostaining for p-tau (Ser202; **B**–**D**), Perls’ Prussian blue staining for ferric iron (**H**–**J**), and immunostaining for MAO-B (**N**–**P**). **E**–**G**, **K**–**M**, **Q**–**S** Scatter plots showing voxel-wise flortaucipir PET SUVR (y-axis) versus CP-13 (**E**–**G**; x-axis), ferric iron (**K**–**M**; x-axis), and MAO-B (**Q**–**S**; x-axis) intensities for each non-AD tauopathy case. Blue lines indicate linear fits (flortaucipir ~ marker), with Spearman’s ρ and *p* values displayed. Abbreviations: CBD, corticobasal degeneration; CP-13, phospho-tau (Ser202) antibody; FTP, flortaucipir; FTLD, frontotemporal lobar degeneration; FTLD-MAPT-S305I, FTLD due to MAPT S305I mutation;; MAO-B, monoamine oxidase B; MRI, magnetic resonance imaging; PET, positron emission tomography; PSP, progressive supranuclear palsy; ROI, region of interest; SUVR, standardized uptake value ratio; WM, white matter. (T) Distribution of MAO-B voxel intensities inside versus outside white matter; Wilcoxon rank-sum *p* value is reported.

Applying the same correlation and regression analyses to PSP, CBD, and FTLD-MAPT-S305I slabs (Fig. 3, Table 2) revealed weak or negligible correlations between Flortaucipir PET and p-tau/CP-13 (PSP: ρ = 0.06, *p* = 0.002; CBD: ρ = − 0.02, *p* = 0.41; FTLD-MAPT-S305I: ρ = − 0.10, *p* < 4.2e−7). Figure 3B–G). Multilinear model m0 confirmed p-tau/CP-13 was not a significant predictor in PSP and CBD and inversely associated in FTLD-MAPT-S305I. AIC values indicated that models including ferric iron and MAO-B better explained Flortaucipir variability, with AIC improvements supporting this (though close differences in FTLD-MAPT-S305I).

In terms of ferric iron (Fig. 3H–M) and MAO-B (Fig. 3N–S), PSP showed no significant Flortaucipir- ferric iron correlation (ρ = 0.008, *p* = 0.67, Fig. 3K), but a weak Flortaucipir-MAO-B correlation (ρ = 0.17, *p* < 2e−16, Fig. 3Q). CBD had weak Flortaucipir correlations with both ferric iron (ρ = 0.15, *p* = 1e−7 Fig. 3L) and MAO-B (ρ = 0.07, *p* = 0.02, Fig. 3R). FTLD-MAPT-S305I exhibited significant Flortaucipir correlations with both ferric iron (ρ = 0.22, *p* < 2e−16, Fig. 3M) and MAO-B (ρ = 0.46, *p* < 2e−16, Fig. 3S).

Overall, p-tau/CP-13’s contribution to Flortaucipir variability was minimal—up to eight times smaller than MAO-B in PSP and inversely associated in CBD and FTLD-MAPT-S305I. Similar to AD, MAO-B burden was concentrated in white matter (Fig. 3T), limiting its impact on cortical Flortaucipir signal. Multilinear models explained 4% and 2% of Flortaucipir variance in PSP and CBD, respectively, but 22% in FTLD-MAPT-S305I.

### FTLD-TDP-43 type A: a non-tauopathy control

**Fig. 4:**
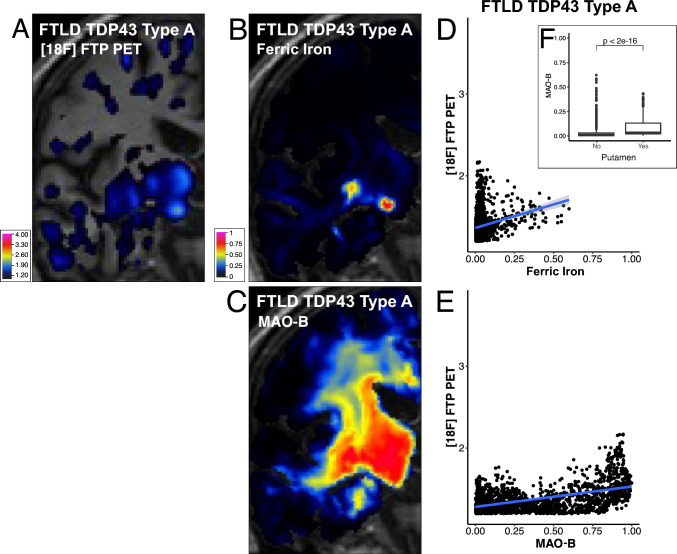
Coronal MRI views of the FTLD-TDP type A Flortaucipir PET data, and their correlation with Ferric iron and MAO-B markers from histological staining data. **A** Flortaucipir PET SUVR map (1.2 [blue] to 4.0 [red]; top) and MRI hemisphere ROI used for analysis (bottom). **B**, **C** Quantitative histological signal (scaled 0–1) mapped to the same MRI space as the flortaucipir PET: Perls’ Prussian blue staining for ferric iron (**B**) and immunostaining for MAO-B (**C**). **D**, **E** Scatter plots showing voxel-wise flortaucipir PET SUVR (y-axis) versus ferric iron (**D**; x-axis) and MAO-B (**E**; x-axis) intensities. Blue lines indicate linear fits (flortaucipir ~ marker), with Spearman’s ρ and *p* values displayed. **F** Distribution of MAO-B voxel intensities within versus outside the putamen; Wilcoxon rank-sum *p* value is reported. Abbreviations: FTP, flortaucipir; FTLD, frontotemporal lobar degeneration; FTLD-TDP-43, FTLD with TDP-43 type A pathology; MAO-B, monoamine oxidase B; MRI, magnetic resonance imaging; PET, positron emission tomography; ROI, region of interest; SUVR, standardized uptake value ratio

To investigate Flortaucipir PET binding independent of tau, we included one FTLD-TDP type A case, with no detectable p-tau/CP-13 signal histologically or via CNN (Fig. 4A, Table 2). Both ferric iron and MAO-B burdens significantly correlated with Flortaucipir PET signal (ferric iron: ρ = 0.33, Figs. 4B–F; MAO-B: ρ = 0.41; both *p* < 2e−16, Fig. 4C, E). In multilinear regression (model m3), MAO-B remained significant (β = 0.24, *p* < 2e−16) while ferric iron was not. Heatmaps showed Flortaucipir intensities were spatially associated with ferric iron accumulation in the putamen (Fig. 4F).

### Investigating flortaucipir PET ON- and off-target signal in the lentiform nucleus

The lentiform nucleus (putamen and globus pallidus) consistently exhibits Flortaucipir retention despite minimal p-tau pathology in AD. We examined correlations between Flortaucipir PET and histological markers in PSP, FTLD-MAPT-S305I, and FTLD-TDP cases, the slabs that contained the lentiform nucleus.

PSP showed a modest positive Flortaucipir- ferric iron correlation (ρ = 0.22, *p* = 0.01). FTLD-MAPT-S305I (ferric iron: ρ = 0.72, MAO-B: ρ = 0.56) and FTLD-TDP (ferric iron: ρ = 0.56; MAO-B: ρ = 0.60) demonstrated positive correlations between Flortaucipir and both ferric iron and MAO-B. Notably, p-tau/CP-13 did not correlate with Flortaucipir in PSP and had only a weak association in FTLD-MAPT-S305I (ρ = − 0.16, *p* value = 0.021).

Multilinear regression using model *m*0 and *m*1 reinforced these findings: ferric iron was the main positive predictor in PSP, and combined ferric iron plus MAO-B models best explained Flortaucipir signal variance in FTLD-MAPT-S305I and FTLD-TDP, while p-tau/CP-13 lacked significant predictive value, as indicated by the AIC (ΔAIC *m*0 vs *m*1 = 0.46).

## Discussion

In this study, our primary goal was to investigate the non-tau contributors to the Flortaucipir PET signal across a spectrum of neurodegenerative diseases. While Flortaucipir is widely utilized as a putative in vivo marker of AD tau pathology, its well-known off-target binding necessitates a comprehensive understanding of other factors influencing its retention. By employing a novel voxel-to-voxel approach that quantitatively relates histopathology data for phospho-tau, ferric iron, and MAO-B with Flortaucipir signal in cases of AD, CBD, PSP, FTLD with a MAPT S305I mutation, and a non-tauopathy FTLD-TDP type A pathology, we sought to clarify the extent to which these non-tau factors influence the Flortaucipir signal. Our method leveraged parallel staining for all three targets (phospho-tau, ferric iron, and MAO-B) within the same individuals and utilized a consistent analytic pipeline across these disease conditions. This approach enhanced the comparability of our findings and minimized potential biases arising from case-by-case methodological differences.

Our analyses in the AD case confirmed previous reports that the Flortaucipir signal is strongly associated with p-tau pathology, supporting its established utility in detecting the characteristic 3R/4R paired helical filament tau aggregates of AD. Post-mortem validation studies have consistently shown that Flortaucipir binds robustly to neurofibrillary tangles in neocortical areas of AD brains [15, 20], correlating with disease severity [3, 31] Yet, even in AD, our results indicated that ferric iron burden contributed modestly to the Flortaucipir signal. This demonstrates that even in a “canonical” disease for Flortaucipir’ s intended target, non-tau substrates can influence tracer retention. Nonetheless, these non-tau effects were relatively small compared to the strong relationship between p-tau/CP-13 burden and Flortaucipir signal in AD, thereby reinforcing the view of Flortaucipir as a reliable marker of tau pathology within this specific disease context.

In non-AD tauopathies, (PSP, CBD, and FTLD-MAPT-S305I), the contributions of p-tau to the Flortaucipir signal diminished considerably. While earlier in vivo imaging studies have suggested that Flortaucipir may detect certain 4-repeat tau pathologies [15, 33], recent postmortem cryo-EM analyses have revealed that tau filaments in these diseases adopt distinct and disease-specific folds and ultrastructures that differ markedly from the paired helical filaments (PHFs) typical of AD. For example, PSP and CBD tau filaments show unique folding patterns with straight filament conformations and specific protofilament arrangements that influence their biochemical properties and likely their tracer binding affinity [30]. This nuanced structural variation highlighted by cryo-EM underscores why Flortaucipir signals in these non-AD tauopathies may reflect a mixture of reduced tau binding and possible off-target interactions rather than straightforward tau pathology detection. A recent large post-mortem autoradiography study showed limited Flortaucipir binding to 4R tau aggregates in PSP and CBD, while demonstrating substantial off-target or non-tau binding [1].

Our findings corroborate prior postmortem work suggesting that, in 4R tauopathies, regional Flortaucipir binding shows limited correspondence with 4R tau aggregates and substantial non-tau binding, underscoring important off-target contributions [1, 9, 20]. Instead, we found that ferric iron and MAO-B contributed to Flortaucipir uptake, offering insights into the sources of off-target signal in these diseases. Notably, the relative influence of ferric iron and MAO-B varied across brain regions and disease types. This variability likely reflects differences in regional substrate abundance, molecular forms, and disease-specific pathology. For example, while strong correlations with ferric iron and MAO-B were observed in FTLD-MAPT-S305I and FTLD-TDP-43 type A cases, such associations were weaker or absent in PSP, indicating heterogeneity in off-target binding substrates within basal ganglia structures. Most importantly, these results suggest that ferric iron and MAO-B do not fully explain all off-target Flortaucipir retention, implying the existence of additional, yet unidentified, binding substrates contributing to tracer uptake.

In the FTLD-TDP case, where tau pathology was essentially absent, our voxel-wise analysis further underscored the predominance of off-target signal. Both ferric iron and MAO-B correlated with Flortaucipir retention in regions not known for tau aggregation, including a strong white matter signal associated with a high burden of MAO-B. Given that MAO-B is markedly up-regulated in reactive astrocytes [7], these strong MAO-B-related FTP signals, particularly in white matter, likely reflect reactive astrogliosis–related off-target binding rather than tau aggregates. While previous studies had reported off-target binding in FTLD-TDP [1, 20], our voxel-to-voxel comparison approach allowed us to directly compare the relative contributions of these non-tau substrates.

The findings from this study align with recent evidence suggesting that the relationship between histological tau pathology and in vivo Flortaucipir is complex and not strictly linear. This highlights the importance of continued investigation into off-target Flortaucipir PET signal, and the present work provides valuable insight into addressing this critical question. Combining Flortaucipir PET imaging with iron-sensitive MRI sequences may be worth exploring in AD.

A significant strength of this study lies in the uniform application of parallel, systematic tissue processing and staining methods across diverse neurodegenerative diseases. By employing the same analytic pipeline to correlate PET signal with p-tau, ferric iron, and MAO-B in each brain, we enhanced the comparability of our findings and mitigated methodological confounds. This consistent approach, combined with voxel-to-voxel comparisons, provides a more nuanced understanding of Flortaucipir tracer behavior and allows for more reliable conclusions regarding off-target binding patterns in different neurodegenerative contexts. Nevertheless, several limitations warrant acknowledgment. Although our analyses generated thousands of voxel-wise comparison points, these arose from a relatively small number of cases, and one with a maximum of two slabs per case, each representing a single snapshot in the context of heterogeneous and complex disease processes. Disease heterogeneity and individual variability remain important considerations when interpreting our findings. For instance, the stratified voxel-wise plots (Fig. S10) show regional differences at similar ferric iron and MAO-B levels. AD regions, especially cortex and hippocampus, exhibit a broader and higher Flortaucipir SUVR range than non-AD tauopathies; PSP and CBD remain low, with FTLD-MAPT-S305I and FTLD-TDP type A intermediate. Combined with the regression models, this indicates that ferric iron and MAO-B influence but do not fully explain regional Flortaucipir differences and other markers warrant testing. Our choice of markers was based on the literature and resources available. Although in vitro studies report higher affinity of Flortaucipir for MAO-A than MAO-B, the in vivo relevance of MAO-related binding remains debated across studies [5, 40]. We prioritized MAO-B endpoints, because of MAO-B enrichment in reactive astrocytes and well-documented MAO-B interactions with another first-generation tau PET ligand [24]. In any case, enzyme inhibition is weak for both isoforms and MAO-A related binding is not expected to significantly affect cortical Flortaucipir signals [1, 41]. The interval between PET imaging and death varied across cases, potentially influencing the correlation strength between imaging and histology. Additionally, comparing histology to PET inherently requires downsampling high-resolution histological data to the coarser PET spatial resolution, which blurs microscopic details and may reduce the sensitivity of voxel-to-voxel correlations. Challenges also remain in standardizing histological quantification across diverse staining protocols and tissue qualities—a process sometimes referred to as "histological normalization", which affects quantitative comparisons.

In conclusion, while Flortaucipir PET reflects p-tau pathology in AD, its interpretation in non-AD tauopathies and FTLD-TDP proteinopathies is considerably more complex. Although our findings confirm that ferric iron and MAO-B contribute to off-target Flortaucipir signal, they do not fully account for all observed tracer retention in these conditions. The insights from our study underscore the importance of identifying additional off-target contributors, refining tracer specificity, and integrating complementary markers to improve diagnostic accuracy and the overall utility of p-tau PET imaging across a broad range of neurodegenerative disorders. For non-AD tauopathies, further research is needed to clarify the histopathological correlations of signals from existing and future PET tracers, and to guide the development of novel ligands tailored to disease-specific tau conformers.

## Supplementary Information

Below is the link to the electronic supplementary material.

Supplementary file1 (DOCX 9402 KB)

## Data Availability

Data available upon reasonable request.
