# Supplementary material for: Disentangling on and off-target binding in flortaucipir PET: a voxel-to-voxel P-tau, ferric iron, and MAO-B histology-to-flortaucipir PET comparison
Source: Acta Neuropathol. 2026 Feb 25;151(1):21. doi: 10.1007/s00401-026-02983-x (PMC12935771; doi:10.1007/s00401-026-02983-x)
Supplement: Supplementary file 1 — Supplementary file1 (DOCX 9402 KB) [file 401_2026_2983_MOESM1_ESM.docx]

**Supplementary Methods and Results**

**INDEX**

**METHODS ........................................................................ 2**

**1.1. Neuropathology methods** ...................................................... **2**
  1.1.1. Tissue embedding and sectioning .................................... **2**
  1.1.2. Ferric iron staining and immunohistochemical staining ........ **2**
  1.1.3. Free-floating immunostaining protocol using monoclonal CP-13 antibody ................................................................. **3**
  1.1.4. Free-floating immunostaining protocol using MAO-B (MAO-B-Rb) antibody ................................................................. **4**
  1.1.5. Perls’ ferric iron staining technique ................................. **5**

**1.2. Digital pathology methods** ................................................ **6**
  1.2.1. Whole-slide imaging .................................................... **6**
  1.2.2. Creation of imaging datasets for IHCNet training and validation ................................................................. **6**
  1.2.3. Deep learning–based segmentation of CP-13 and MAO-B histological signal: model development and training ............... **8**
  1.2.4. Segmentation and quantification of ferric iron ............... **9**
  1.2.5. Pre-processing blockface images ................................ **10**
  1.2.6. Low-resolution histology background segmentation ........ **11**
  1.2.7. 2D registration to blockface images .......................... **11**
  1.2.8. Image and mask tiling .............................................. **12**
  1.2.9. Heatmap computation ............................................... **12**
  1.2.10. Heatmap to MRI alignment and registration ............. **13**

**SUPPLEMENTARY FIGURES**

**Fig. S1.** Complete study pipeline ............................................. **14**
**Fig. S2.** Representative coronal hemisphere slabs and histology from the five neuropathologically confirmed cases included ......................................................... **15**
**Fig. S3.** Components of the in-house–built large-slide scanner ........ **16**
**Fig. S4.** Workflow from histology images to quantitative segmentation maps ................................................................. **17**
**Fig. S5.** IHCNet architecture ..................................................... **18**
**Fig. S6.** Training performance of the CNN for CP-13 (p-tau) and MAO-B segmentation ................................................................. **19**
**Fig. S7.** Ferric iron model performance and segmentation ............ **20**
**Fig. S8.** 3D reconstructions and histology/MRI/PET heatmaps .... **21**
**Fig. S9.** Dice coefficient analysis of registration accuracy between histological slabs and MRI ................................................................. **27**

**RESULTS ........................................................................ 28**

**Fig. S10.** Voxel-wise distributions of Flortaucipir SUVR and histological signals across ROIs ................................................................. **28**
**Table S1.** Spearman correlations between Flortaucipir PET SUVR and histological signals under different SUVR thresholds ........ **30**

**REFERENCES ................................................................. 30**

**METHODS:**

**Neuropathology Methods**

Most of the pipelines were tested and published in detail previously [1] and Fig S1. The most important parts of the protocols and modifications are also described below.

**Tissue embedding and sectioning**

After fixation, the selected slab for each case was embedded in celloidin. The slabs were mounted and sectioned in serial 160 μm-thick sections using a sliding microtome with a C-shaped knife. The section thickness was selected to balance the risk of tissue tears and the efficiency of antibody penetration. During sectioning, digital photographs were acquired directly from the blockface following each stroke using a high-definition, computer-controlled DSLR camera (EOS 5D Mark II, Canon, Tokyo, Japan) mounted on a copy stand arm (Kaiser Fototechnik, Germany). Details of tissue processing, celloidin embedding and cutting have been described by us elsewhere [1, 2].

**Ferric iron staining and Immunohistochemical staining**

Free-floating staining reactions were performed including positive and negative controls consisting of serial sections from a single non-study subject. Batches in which the positive control exhibited low-quality staining were excluded, and the staining procedure was repeated on the next set of contiguous tissue sections. To avoid signal mixing, all staining was conducted using single-label immunohistochemistry, and stained sections were subsequently mounted on 6" × 4" glass slides.

For immunohistochemical mapping, we generated 3D histological series using two widely used phospho-tau antibodies (p-tau; 1:400, CP-13 [p-tau Ser202], mouse, gift from Peter Davis; developed with Impact DAB). Parallel sections were immunostained for MAO-B (1:1800, HPA002328, rabbit, Sigma-Aldrich; developed with Impact DAB) or processed for ferric iron staining using Perls’ method. Within each set of serial sections, the first section was stained with CP-13, the second section from even-numbered sets was processed for ferric iron, and the third section from even-numbered sets was stained for MAO-B.

Protocols are reproduced below:

| **Free Floating Immunostaining Protocol Using Monoclonal CP-13 Antibody**  **100µm – 160 µm Brain Slabs, Cerebellum OR Whole Brain Sections**  **Day 1: (dehydrate section from 80% EA 1x30min, then 96% EA overnight-no agitation)**   1. Quench sections in 3% H2O2/Methanol for 40min – slow agitation. 2. Rinse in distilled water for 3x3min each 3. Place sections in labeled filter paper; Seal pouch with staples. Ensure pouch is filled with AR buffer and section is floating inside. 4. Place pouches in working 0.01M citrate buffer/ distilled water /0.5% Triton X-100 pH6. Autoclave at 250F for 5min. 5. Let sections cool at RT for 30min 6. Wash in PBS/0.5% Triton 2x8min each 7. Block sections in 5% Milk/PBS 0.5% Triton for 40min 8. Incubate all sections in **monoclonal CP-13** (gift from Peter Davies Einstein Institute **stock 1:5**) & **dilute at 1:400 using 5% Milk/PBS/0.5% Triton – at 4C for 2 days.**   **Day 3:**   1. Wash in PBS/0.5% Triton 2x8min each 2. Incubate in biotinylated anti-Ms at 1:400 in PBS 0.5% Triton for 1.5 hours 3. Wash in PBS/0.5% Triton 2x8min each 4. Incubate in ABC-HRP for 1.5 hours (ABC-HRP needs to be made 30min before use) 5. Wash in PBS/0.5% Triton 2x8min 6. Develop in Impact DAB (prepare per manufacturer’s instructions) 7. Stop development in distilled water 8. Wash in PBS-Azide 2x8min each and store at 4C until ready for mounting. 9. Mount section to microscope slide. Let section dry overnight without filter paper and have slides stand vertically on slide rack. Coverslip with in-house made Permount glue the next day. |
| --- |

| **Free Floating Immunostaining Protocol Using Monoclonal MAO-B Antibody**  **100µm – 160 µm Brain Slabs, Cerebellum OR Whole Brain Sections**  **Day 1: (dehydrate section from 80% EA 1x30min, then 96% EA overnight-no agitation)**   1. Decelloidinize brain slabs: 1:1 ether/100%EA 2x3min, 100% EA 2x5min, 96% EA 1x15min 2. Quench sections in 3% H2O2/Methanol for 40min 3. Rinse in distilled water 3x3min 4. Place sections in labeled filter paper; seal pouch with staples 5. Places pouches in working 0.01M citrate buffer/ distilled water /1% Triton pH6. Autoclave at 250F for 5min 6. Wash in PBS/1% Triton 2x8min each 7. Block sections in 5% milk Powder/PBS 2% Triton for 40min 8. Incubate in MAO-B-Rb (Stock FS) at 1:1800 at 4C overnight    1. 8.3ul of MAO-B-Rb + 15ml of diluent   **Day 2:**   1. Wash in PBS/1% Triton 2x8min each 2. Incubate in biotinylated anti-Rb at 1:400 for 1.5 hours    1. 50ul of biotinylated anti-Rb + 20ml of diluent 3. Wash in PBST 2x8min 4. Incubate in ABC-HRP for 1.5 hour (ABC-HRP needs to be made 30min before use) 5. Wash in PBST 2x8min 6. Develop in Impact DAB. Stop development in distilled water 7. Wash in PBS 2x8min each and store at 4C until ready for mounting. 8. Let section dry overnight with filter paper and bulkhead nut on top. Coverslip with in-house made Permount glue the next day. |
| --- |

| **Perls’ Ferric Iron Staining Technique**  **100µm – 160 µm Brain Slabs, Cerebellum OR Whole Brain Sections**  ***Make fresh Perls’ Iron staining solution for each run***  **Solution A**  **Potassium Ferrocyanide** – **2g** (synonym **K. hexacyano ferrate (II) trihydrate** ACS chemical salt)  (Order from Sigma/Aldrich # P3289-100G)  distilled water – 100ml  Dissolve & add:  **Concentrated hydrochloric acid** – **2ml**  (Use reagent-grade HCl (reagent grade which does not contain iron.)  **Solution B**  Counterstain for nuclei  **0.1%** aqueous **Nuclear Fast Red + 5% Aluminum Sulfate**  **Day 1:**   1. Cut celloidin excess close to tissue edge (NO need to dehydrate sections). 2. 60µm sections are stored in **70% EA** - just rinse in **distilled water** 2X – 1 minute total. 3. Immerse in freshly made **acid ferrocyanide** (**solution A**) for **15 minutes**. 4. Wash well in distilled water **4X** – **1 minute each**. 5. Counterstain nuclei (**solution B**) using **Nuclear Fast Red** – **2 minutes.** 6. Wash sections briefly in large petri dish with distilled water – rinse each section a few seconds. *Change distilled water as needed. 7. Mount sections using pre-labeled regular sized glass slides & flatten using filter paper.   Place metal bolts on top of section to flatten tissue & store inside dry desiccator box.  **Day 2:**   1. Cover slip sections using **special mounting media for thick sections**.   **Results:** Blue precipitate with Fe+ liberated from ferritin & hemosiderin. Nuclei - either pink or red. |
| --- |

Fig S2 shows images of the block face, stained sections and magnifications highlighting CP-13, iron, and MAO-B positive signals in high magnification.

**Digital pathology methods**

**Whole-Slide Imaging**

We built whole-slide scanner to accommodate our histological slides (4" x 6"), which do not fit into regular microscope stages or cannot be fully imaged due to short stage travel range (**Fig. S3)**. The hardware comprises a high-precision, 6" travel range, industrial XY stage (Griffin Motion), an Olympus manual focusing box, color CCD camera (Qimaging Micro publisher 6), and 5.5X machine vision objective (Navitar Zoom 6000) mounted directly on the camera. Illumination is performed by a lightbox with diffuser, mounted on top of the XY stage. Sections were loaded directly to a 3D printed slide mount fixed on top of the lightbox. At 6.75x magnification, the objective field of view was 3.28 x 2.6 mm. The scanner was controlled by software developed in-house using Macro Manager 2.0^42^, which has a user interface that allows defining the region-of-interest **(ROI)**, performs white balance, and select lens magnification parameters. Macro Manager 2.0 computes the image tiles coordinates necessary to cover the selected ROI and synchronizes the XY stage movements with image capture. TeraStitcher [3], which can work with several Gigabytes of data while maintaining a small memory footprint were used to stitch full resolution tiles (1.22μm/ pixel resolution) and create histological images of each slide. A 10% resolution version of each slide image was also created during the stitching process for visual quality control and aiding with histology pre-processing and registration steps. Our scanner software can be downloaded at (<https://github.com/grinberglab/high-res-3D-tau>).

**Creation of imaging datasets for IHCNet training and validation (Fig S4)**

We generated 1024x1024 pixels (1.25 x 1.25 mm) patches from randomly selected gray matter locations throughout the full-resolution brain image datasets to create training, testing, and validation datasets. Before patch extraction, we applied manually segmented white matter masks to the full-resolution brain image datasets. This step aimed to generate training and validation datasets enriched for gray matter patches (because tau pathology is predominantly located in gray matter in AD). However, we did not mask for MAO-B dataset since MAO-B signal is not restricted in gray matter. We generated 1024×1024 pixels (1.25 × 1.25 mm) patches from randomly selected gray matter locations throughout the full-resolution brain image datasets to create training, testing, and validation datasets. Before patch extraction, we applied manually segmented white matter masks to the full-resolution brain image datasets (5 cases). This step aimed to generate datasets enriched for gray matter patches (because tau pathology is predominantly located in gray matter in AD). However, we did not mask for the MAO-B dataset since MAO-B signal is not restricted to gray matter. The patch extraction routine was written in Python and completely automated, running on UCSF's Wynton cluster (https://wynton.ucsf.edu), exploring computational parallelism while extracting patches, i.e., the different images are split into patches simultaneously. For CP-13 (p-tau), the training dataset comprised 254 (1024 × 1024 pixels) randomly selected, expert-labeled p-tau images; these training images were augmented with stride-tiling (stride-step-size = 90 pixels), resulting in 36,576 training dataset images. In a similar pipeline, the MAO-B training dataset contained 38,448 labeled images. Each patch was manually masked for background and p-tau/MAO-B inclusion with the help of Fiji’s Trainable Weka Segmentation plugin ([www.cs.waikato.ac.nz/ml/weka](https://www.cs.waikato.ac.nz/ml/weka)) [4]. Here, the user manually selected sample pixels belonging to p-tau or MAO-B (depends on the images one is working with) and background classes. These pixels were used to compute Gaussian filters, Hessian, membrane projections, mean, maximum, anisotropic diffusion, Lipschitz, Gabor, Laplacian, entropy, Sobel, a difference of Gaussians, variance, minimum, median, bilateral filter, Kuwahara, derivatives, structure, and neighbor values. A linear SVM classifier (LibLINEAR) was then used to generate an initial tau segmentation [5] . First, a user retrained and refined the initial segmentation using an image editor (Gimp) (<https://www.gimp.org>). Next, all final masks went through quality control by an experienced pathologist. Labeling took approximately 1 hour per patch (total 400h or 50 days of specialized work).

Finally, patches for each antibody (CP-13 and MAO-B) were randomly split into 80% of patches for training, 10% for testing, and the remaining for validation.

**Deep learning-based segmentation of CP-13 and MAO-B histological signal: Model development and training**

IHCNet is a home-developed U-Net [6] based neural network capable of working with various pixel information and outputting tau or MAO-B presence confidence maps that were later thresholded to create binary maps. Our model training pipeline has a 204x204x3 pixel input layer to accommodate RGB images. This patch size, representing approximately 0.06 mm^2^ (249 x 249 um) at our 1.22 um/pixel resolution, is chosen to provide sufficient biological context for p-tau and MAO-B inclusions while remaining computationally efficient for GPU memory. **Fig. S5** shows the IHCNet architecture together with each layer tensor size. The image is pushed through three contractions blocks, the bottleneck, and unsampling by three expansion blocks in our model.

Each contraction block comprises two convolution layers that use 3x3 kernels, stride of 1 and ReLu activation, followed by a 2x2 max pooling layer and a 0.1 rate dropout. The bottleneck comprises two convolutional layers that use 1x1 kernels, stride of 1, and ReLu activation. Expansion blocks are composed of a 2x2 upsampling layer followed by a 0.1 rate dropout and two convolutional layers that use 3x3 kernels, stride of 1 and ReLu, except for the last expansion block that uses 3x3 upsampling. The last layer reshapes the data to a 20000x2 tensor and softmax activation.

Training was performed using standard backpropagation, with binary cross-entropy as the loss function and Adam algorithm for optimization. The learning rate is estimated using the cyclical learning rate method [7]. The network was developed in Keras (<https://keras.io>) on top of TensorFlow (<https://www.tensorflow.org>). We trained the network using mini-batches of 32 images for 100 epochs or until the loss curve plateaued. We also used massive data augmentation, performing real-time random rotations, shear, horizontal and vertical flips. All training and inference were performed on an NVIDIA Titan V GPU with 12 GB of RAM. IHCNet outputs confidence maps of the existence of tau inclusions, which are thresholded to generate binary masks. For both CP-13 and MAO-B, the probability threshold was set to 0.7, chosen based on precision–recall curve analysis and neuropathologist visual review; pixels with confidence ≥ 0.7 were considered positive and those < 0.7 as background (Fig. S6). IHCNet training took up to 2 days per antibody using a workstation with 2 GPUs. The networks were trained until we observed a plateau in the accuracy and loss function graphs.

**Segmentation and Quantification of Ferric Iron**

Because Perls’ ferric iron staining produces substantially lower background noise than CP-13 or MAO-B immunostaining, segmentation did not require the sophisticated deep-learning approach used for the immunohistochemical markers. For training on ferric iron segmentation, we use Trainable Weka Segmentation (TWS) plugin in Fiji ([www.cs.waikato.ac.nz/ml/weka](https://www.cs.waikato.ac.nz/ml/weka)) [4] with LibLINEAR classifier [5] to create a machine-learning model (**Fig. S7**). Ferric iron staining with Perls’ blue, compared to p-tau and MAO-B immunostaining, generally has less complex background noise, and exhibited stable, uniform chromogenic properties suitable for TWS machine learning based classification. The training processes are the following: 1. We randomly select 100, 2688x2200 pixel tiles from ferric iron scanning, and split them into 80% for training data and 20% for testing data (there is no validation dataset because of the fundamental difference between TWS and CNN model training pipeline, TWS have an internal algorithm for validation dataset generation instead of user input validation dataset like CNN). 2. We input each of the training tiles to TWS plugin in serial fashion, trained lab technicians will sample ferric iron pixels as positivity class and background pixels as negative class with Fiji’s ROI selection tool with the first tile and start the model training, trained results included model performance statistic and iron segmentation based on the current model. 3. This segmentation result will be visually reviewed/inspected and further refined with additional ROI sampling. Once the iron model segmentation result and ground truth (manually traced ferric iron segmentation by trained lab technicians) has mIoU higher than 0.75 (mean Intersection over Union, indicate how well the model segmentation correspond to ground truth, 1 mIoU indicate perfect segmentation), we would then save the data from TWS, load it to the second tiles and train, then continue to review the result and refine. This process will accumulate training data from the entire training dataset (in arff format), and the product is the model file that we will use to generate ferric iron segmentation. 4. We further validate the model performance by computing two metrics like our CNN IHCNet training: Precision/Recall (PR) curve and Receiver Operating Characteristic (ROC) curve. The ROC area under curve for both training and testing dataset are similar and above 0.85 (0.93 for training dataset and 0.86 for testing dataset) indicates good discrimination performance in ferric iron segmentation. We used the Precision/Recall curve for deterministic threshold calculation of the ferric iron probability map, and the optimal threshold is set to 0.93 based on F1 score. 5 (**Fig. S7**). This finalized model and segmentation threshold will apply to all ferric iron staining images to generate quantitative heatmap to estimate ferric iron in 1mm^2 resolution, like CP-13 and MAO-B heatmap calculation.

**Pre-processing blockface images**

Blockface images had their background segmented using a semi-automated graph-based algorithm. Briefly, the user selects brain and background sample pixels using a graphical user interface (GUI). Images are then converted to LAB color space (L* for perceptual lightness, and a* and b* for the four unique colors of human vision: red, green, blue, and yellow), and the algorithm computes mean color difference maps (ΔE). ΔE is defined as the distance in LAB space:


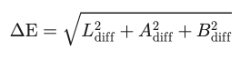


With *L_diff_*, *A_diff_*, *B_diff_* being the difference values computed as:


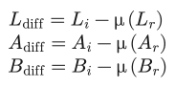


Where *L_i_*, *A_i_* and *B_i_* are image LAB channels, *L_r_*, *A_r_*, *B_r_* are LAB channels of reference pixels selected by the user, and μ(.) is the mean. Pixels in ΔE, whose color is similar to the reference values, appear dark (smaller distance) while cell pixels are brighter (larger distance). We computed brain and background ΔE maps using the manually selected pixels as reference values and performed a global histogram threshold using the Otsu's method to obtain binary masks. We, in turn, combine both masks to obtain brain segmentation. It is expected that several undesired objects linger after the initial segmentation. The segmentation is further refined using a graph-based method to remove the undesired objects. In this method, image objects and their relationship are modeled as a weighted graph, where connected structures are considered the vertices. Edge weights are computed using a similarity function computed from color and distance values. The graph is partitioned using NCuts [8], leaving just the brain area. Commonly, the camera or brain must be repositioned several times during sectioning to adjust for changes in block size, causing the blockface images to be misaligned in relation to each other. We used Matlab's registration GUI to select landmarks for computing affine registrations. MATLAB The MathWorks Inc, Natick, Massachusetts.) Finally, the aligned blockface images were stacked together to form the blockface 3D volume using Insight Segmentation and Registration Toolkit (ITK) [9]. This 3D volume was used as an intermediate space for mapping quantitative, histological heatmap to MRI and PET.

**Low-resolution histology background segmentation**

The 10% resolution histological image datasets were converted to LAB color space. In that space, background pixels are consistently darker than brain pixels, and segmentation is performed through histogram thresholding using the triangle algorithm [10]. The resulting binary masks are used to erase all background pixels. Moreover, brain masks are combined with white matter masks to create gray matter masks that guided the entire segmentation process.

**2D registration to blockface image**

After background segmentation, the 10% resolution histological images are aligned to their respective blockface images using a combination of manual and automatic registration. Due to an excessive number of artifacts caused by histological processing, we initialized the registration manually using MIPAV spline-based registration [11]. Here, the user manually selects landmarks on both the histology and blockface images. MIPAV then generates a warped image and a registration warp file. After the initial registration, the image went through a diffeomorphic registration using the 2D SyN algorithm based on the large diffeomorphic deformation model metric mapping (LDDMM) method [12].

**Image and Mask Tiling**

Each full-resolution histological slide image was first tiled to reduce memory footprint during image segmentation, with tile size corresponding to approximately 5mm^2^ of tissue. Tile coordinates and dimensions are saved as XML metadata files. The histological images’ respective 10% resolution tissue mask (whole image minus the background outside of the tissue) are rescaled to match their full-resolution dimension and tiles. Finally, histological tiles were masked using their respective tissue image tiles, leaving only the ROIs for image segmentation. Tiles with less than 5% of tissue pixels were ignored during segmentation to reduce the overall computational time. Image and mask tiling routines were developed in Python [13] and ran on UCSFs' Wynton cluster exploring computational parallelism, having one pipeline for each histological image.

**Heatmap computation**

The binary tiles were transferred back to the cluster for computing heatmaps. (**Fig. S1**). We computed the mean amount of p-tau, ferric iron, and MAO-B on each tile, indicated by the mean number of pixels belonging to the foreground within a 0.1 x 0.1 mm area of tissue (an 82 x 82 pixel block at 1.22 µm/pixel resolution). The heatmaps were generated as tiles having the same dimensions as the binary tiles, where each 0.01 mm^2^ block is filled with the mean staining positivity. Tiles were then stitched and resized to 10% resolution to match the estimated 1.0 mm T1-MRI voxel size.

**Heatmap to MRI alignment and registration**

The 2D registration matrixes computed during the 2D registration of histology images to blockface images. These matrixes were applied to each corresponding 10% resolution normalized heatmap, yielding heatmap registered to their respective blockface images. As shown in Figs. S1h and S7, by using 3D blockface images that are registered to 3D MRI images as the anatomical reference, we locate the coronal region in MRI from each case that matches our histological tissue, by mapping them in Freeview [14] with rigid transformations. We then extract the matching MRI slides at coronal plane for each stained tissue slides/blockface, and perform 2D registration again with MIPAV and ANTs registration tool to register the histological heatmap to MRI in voxel-to-voxel fashion. Registration performance is calculated with Dice coefficient between 2D registered blockface/histology and T1-MRI hemisphere (**Fig S9**). Since all staining slides from each case are neighboring slides and 160 micrometers in thickness, each set of p-tau/ferric iron/MAO-B heatmap will register to each corresponding MRI slide (approximately 1 mm in thickness). These registered heatmaps will run statistical analysis with corresponding Flortaucipir PET coronal slides (again, PETs are registered to MRIs, thus the same slides-extraction information used for MRI also apply to PET as well), details are described in Method section.

**Supplementary Methods Figures**

**Fig. S1 *Complete study pipeline****.* (a) T1-weighted MRI preprocessing with field correction and background segmentation. (b) Tissue processing with fixation, celloidin embedding, and coronal brain cutting (serial hemisphere sections, 160 µm thick); blockface images are acquired for each section. (c) Blockface image processing with background removal, anterior–posterior axis alignment, and conversion to NIfTI format with 3D stacking. (d) Immunostaining of hemisphere sections in the order p-tau/CP-13, Perls’ ferric iron, and MAO-B for every third serial section. (e) Digitization of stained tissue using an in-house large-slide scanner (spatial resolution 1.22 µm/pixel) and image-tile stitching. (f) Segmentation pipeline using a convolutional neural network (IHCNet) for p-tau/CP-13 and MAO-B, and a machine-learning classifier (LibLINEAR in Trainable Weka Segmentation) for ferric iron, yielding quantitative histology heatmaps normalized from 0 to 1 within ~1 mm² regions. (g–h) 2D and 3D registrations with MIPAV, ANTs, and FreeSurfer/Freeview to map blockface, histology, and heatmaps to T1-MRI, and subsequently to Flortaucipir (FTP) PET for statistical analysis.


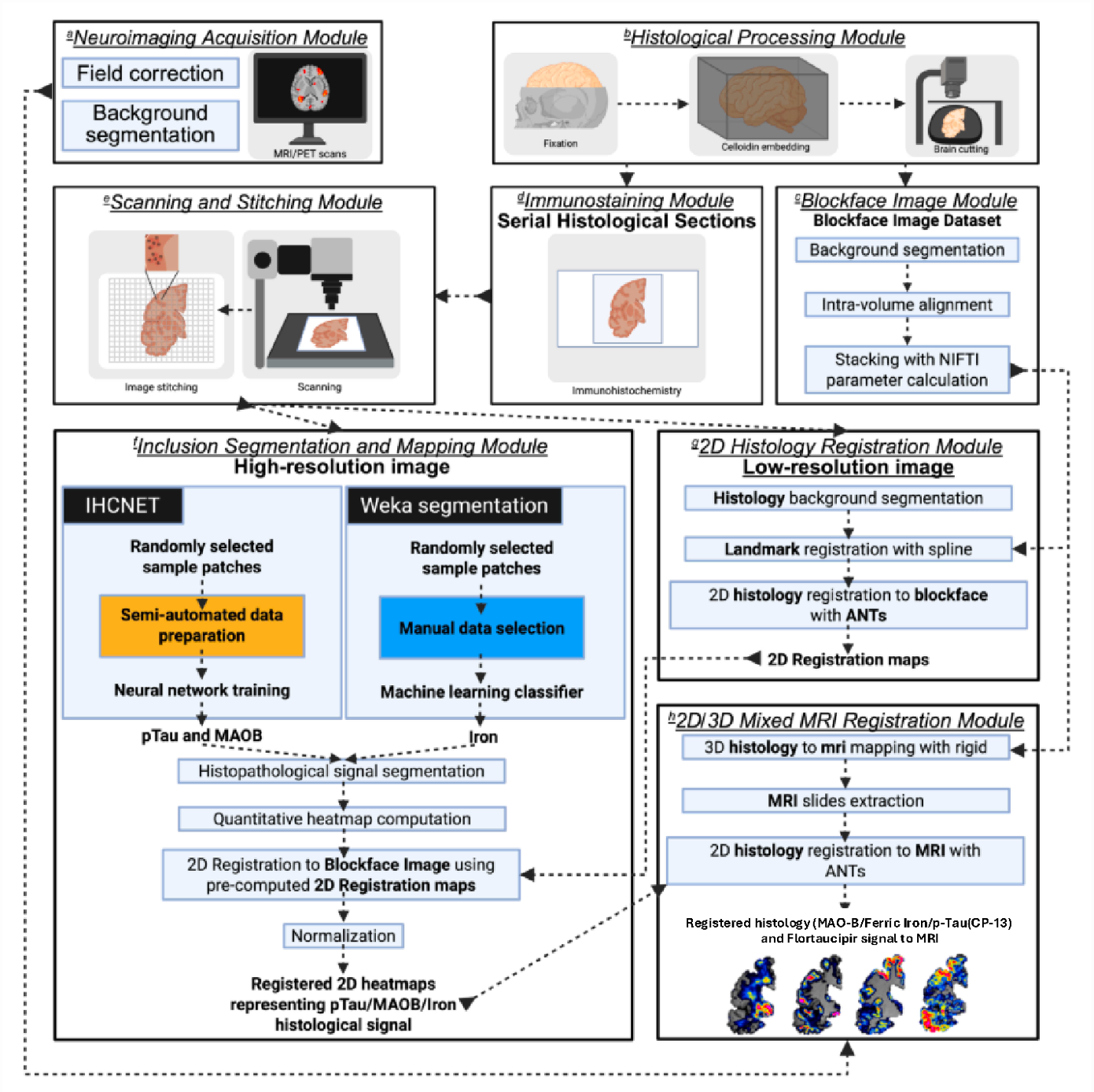


**Fig S2. Representative coronal hemisphere slabs and histology from the five neuropathologically confirmed cases included in this study:** Alzheimer disease (AD), progressive supranuclear palsy (PSP), corticobasal degeneration (CBD), frontotemporal lobar degeneration with MAPT S305I mutation (FTLD MAPT S305I), and FTLD TDP-43 type A. The top row shows blockface images of each slab. For every case, low- and high-magnification images of p-tau/CP-13 immunohistochemistry (rows 2–3), ferric iron staining (Perls’ method; rows 4–5), and MAO-B immunohistochemistry (rows 6–7) are shown. White arrows indicate examples of positive p-tau, ferric iron, or MAO-B signal. Note the absence of p-tau/CP-13 immunoreactivity in the FTLD TDP-43 type A case compared with the tauopathies (AD, PSP, CBD, and FTLD MAPT S305I). Scale bars in high- magnification panels as indicated.


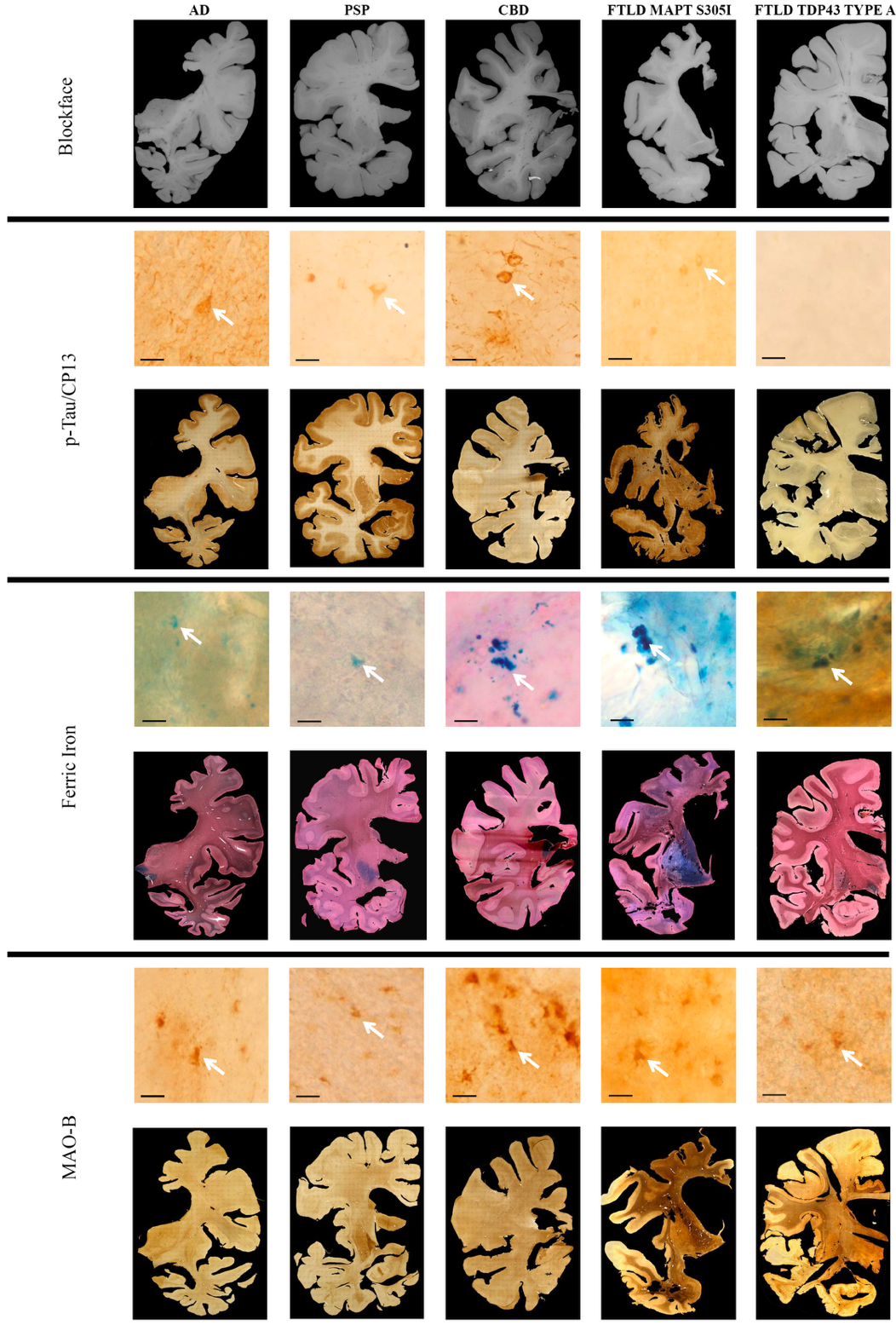


**Fig. S3 Components of the in-house large-slide scanner**. Photograph of the motorized XY stage, lightbox and diffuser, 3D-printed slide holder, camera, and objective used to digitize 4″ × 6″ histological slides.

**
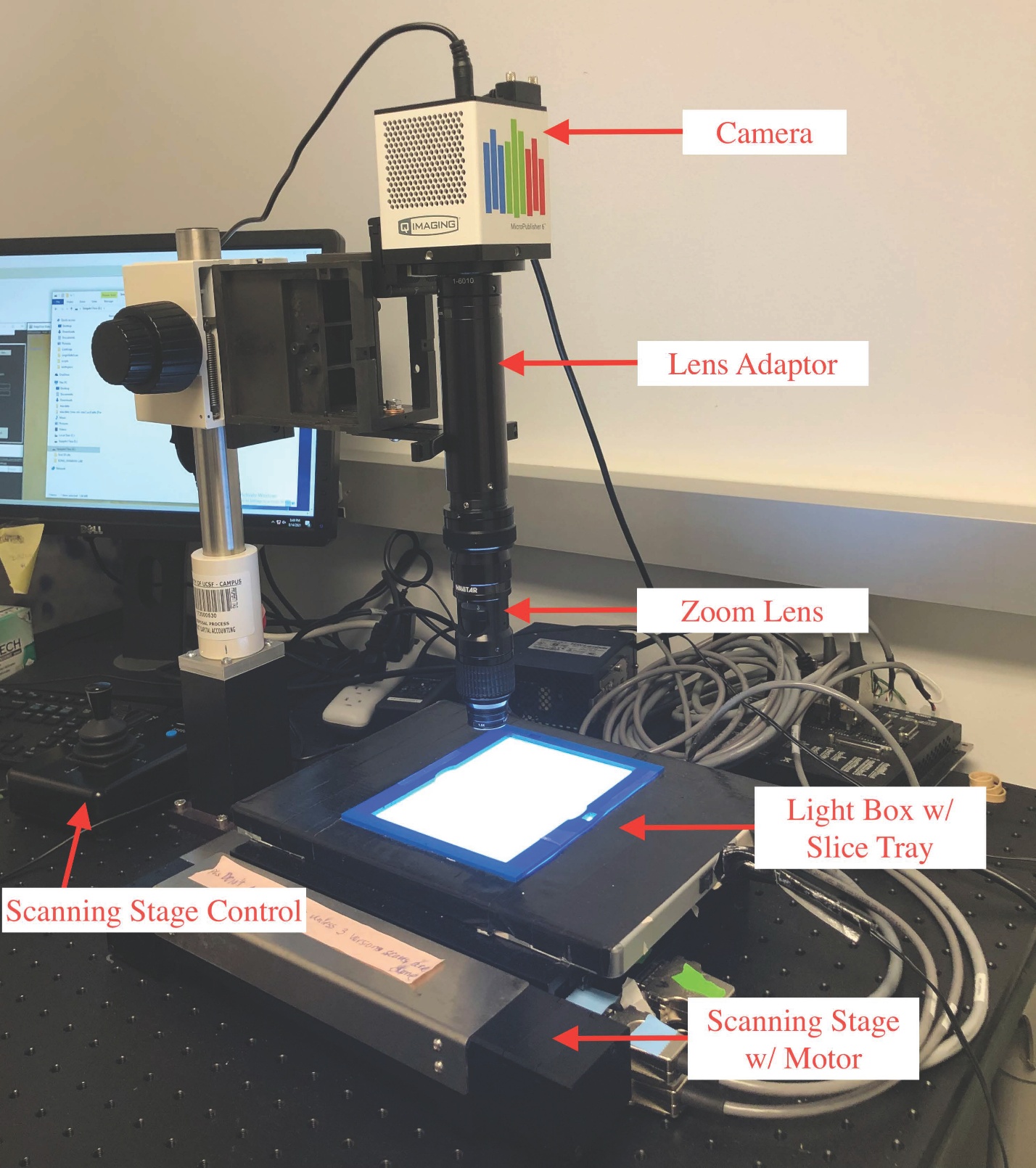
**

**Fig. S4 *Workflow from histology images to quantitative segmentation maps.*** Example from an AD corpus callosum coronal section showing (from left to right): the scanned histological image; CNN- (IHCNet) and ML- (LibLINEAR) generated probability maps (values 0–1) for p-tau/CP-13, MAO-B, and ferric iron; deterministic quantitative segmentation maps after thresholding using optimized precision–recall statistics; and the corresponding histological signal in the highlighted regions (red circles). **
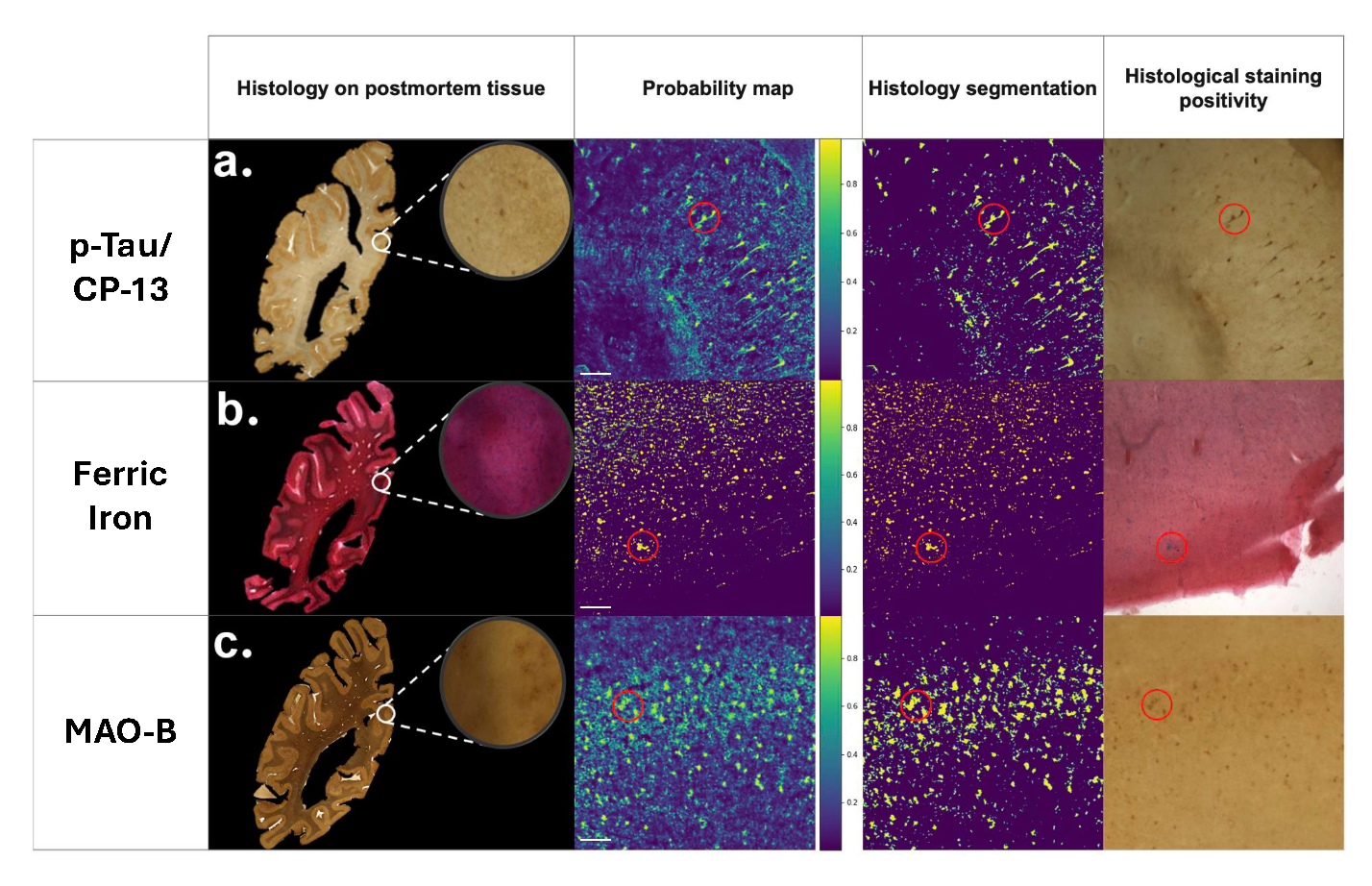
**

**Fig. S5 *IHCNet architecture.*** Schematic of the U-Net–like CNN used for histology segmentation. The dataflow progresses from left to right through contraction blocks, a bottleneck, and expansion blocks. Arrows denote skip connections; in cases where the feature map sizes differ, the larger feature map is cropped to match the smaller one before concatenation.

**
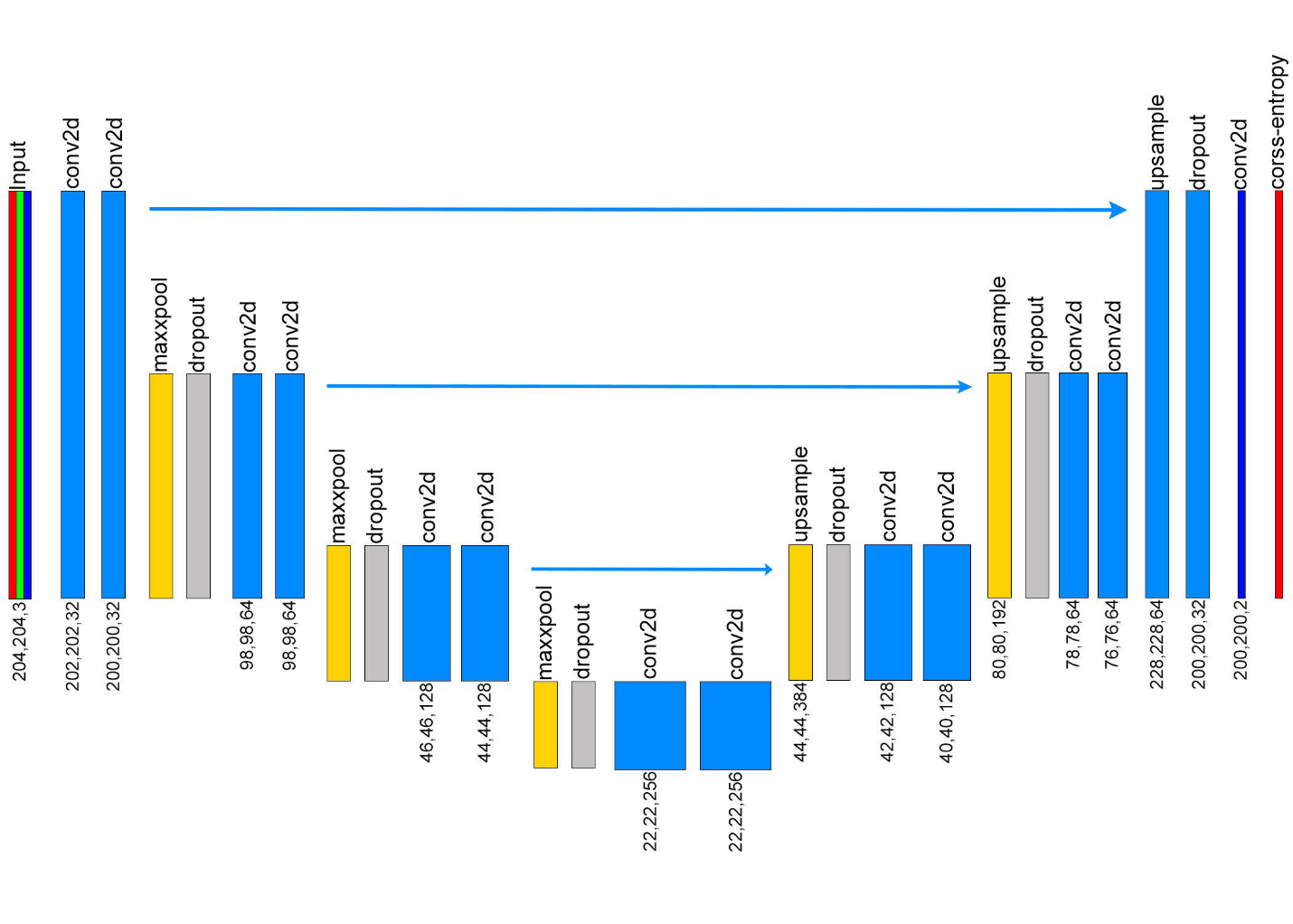
**

**Fig. S6 Training performance of the CNN for CP-13 (p-tau) and MAO-B segmentation.** Panels (a) and (c) show precision–recall curves for the p-tau and MAO-B models, respectively. The area under the precision–recall curve (AUC-PR) for the testing and validation sets was 0.64 and 0.71 for p-tau, and 0.62 and 0.69 for MAO-B. Panels (b) and (d) show the corresponding receiver operating characteristic (ROC) curves; the area under the ROC curve (AUC-ROC) was 0.80 and 0.81 for p-tau, and 0.80 for both testing and validation for MAO-B. Panels (e) and (h) illustrate CNN-derived probability maps indicating the likelihood of positive p-tau and MAO-B staining. Panels (f) and (i) show the thresholded probability maps (threshold = 0.7) used for downstream analysis, and panels (g) and (j) show the original immunohistochemistry images. Scale bar: 50 µm.

**
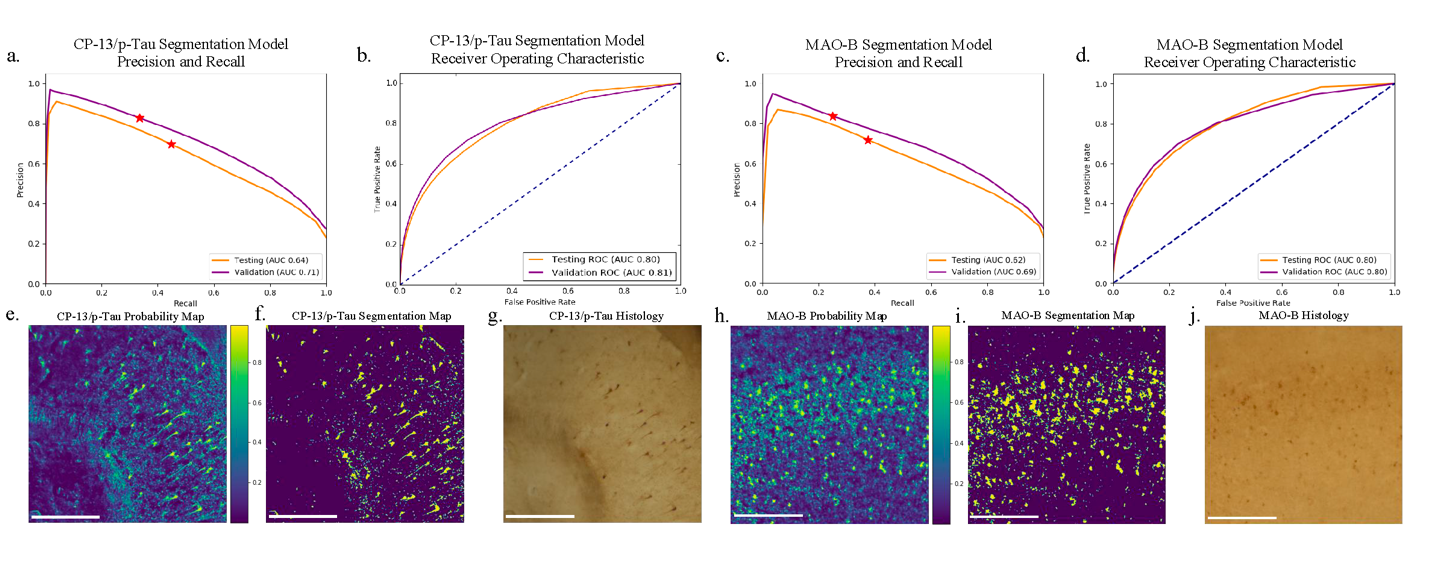
**

**Fig. S7 *Ferric iron model performance and segmentation.*** (a) Receiver operating characteristic (ROC) curve showing good discrimination of the ferric iron model (AUC = 0.93 for the training dataset and 0.86 for the testing dataset). (b) Precision–recall curve used to determine the segmentation threshold. (c) Original ferric iron staining image. (d) Manual tracing of ferric iron, used as ground truth. (e) Machine-learning segmentation generated by the trained model. (f) Visualization of the intersection over union (IoU) between ground truth and model prediction, highlighting true positives, false positives, and false negatives; mIoU denotes the mean IoU score. Scale bar: 40 µm.

**
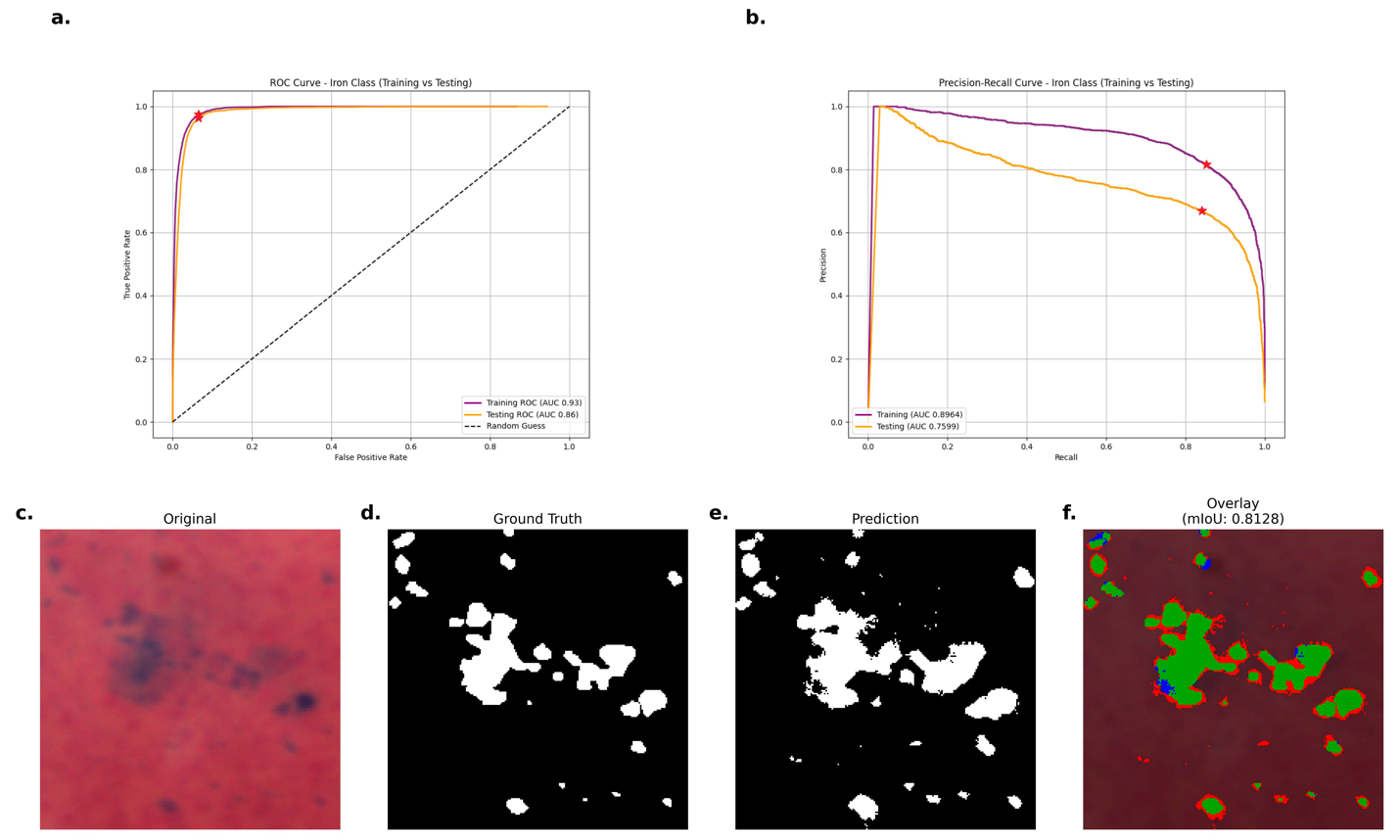
**

**Fig. S8 *3D reconstructions and histology/MRI/PET heatmaps.*** For each case, the top left panel shows the 3D hemisphere blockface reconstruction after alignment along the anterior–posterior axis, resampling to T1-MRI resolution, and rigid + ANTs registration. The top right panel shows the corresponding T1-weighted MRI after skull stripping, hemisphere extraction, rigid registration, and intensity-based background removal. The middle panel overlays the registered blockface and T1-MRI volumes to guide extraction of matched coronal MRI/Flortaucipir PET slices (blockface voxels are displayed with a PET-like color scale for illustration). The bottom panels show registered 2D blockface, histology (p-tau/CP-13, ferric iron, and MAO-B; p-tau/CP-13 not shown for the FTLD TDP-43 type A case), and Flortaucipir PET heatmaps on MRI used for statistical analyses.

**
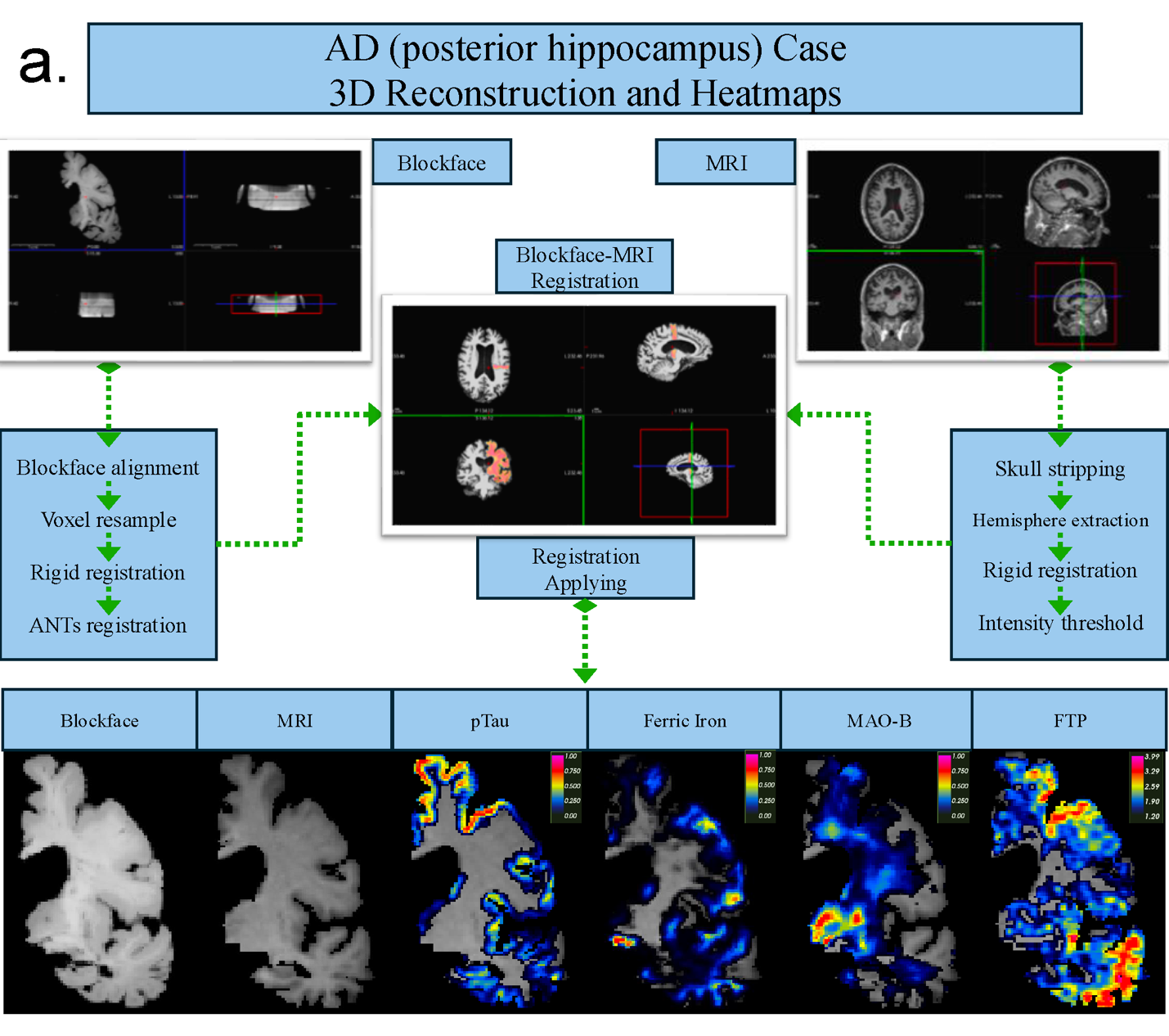
**

**
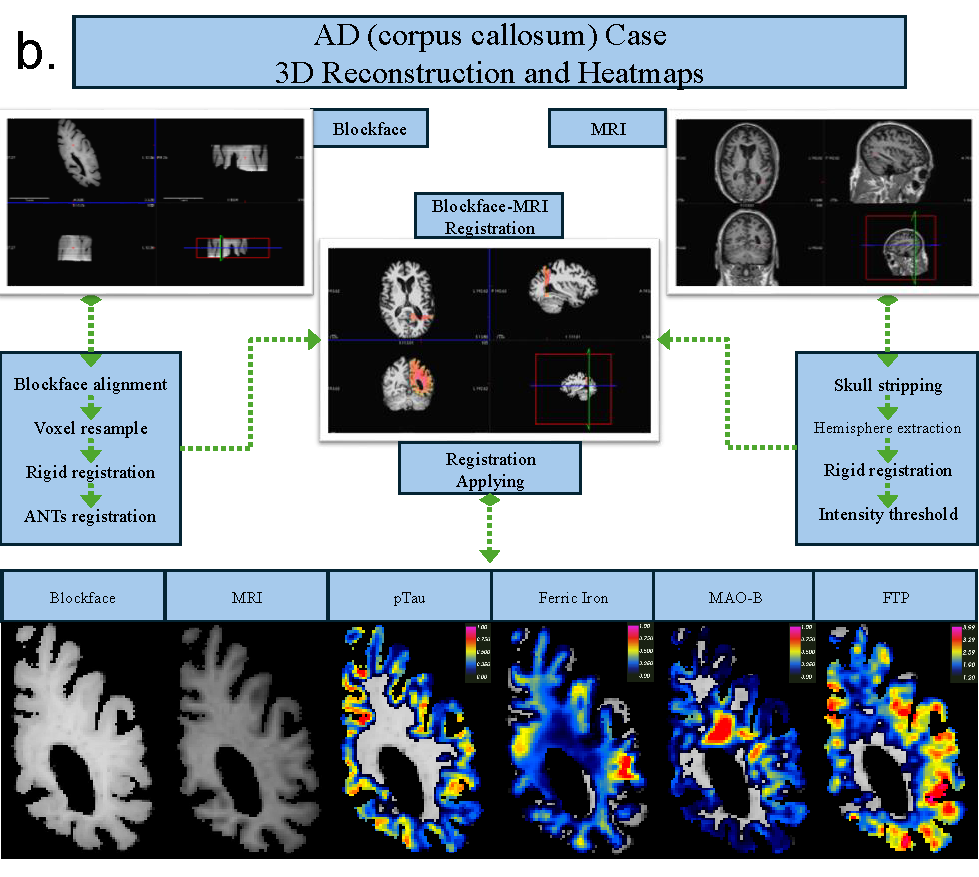
**

**
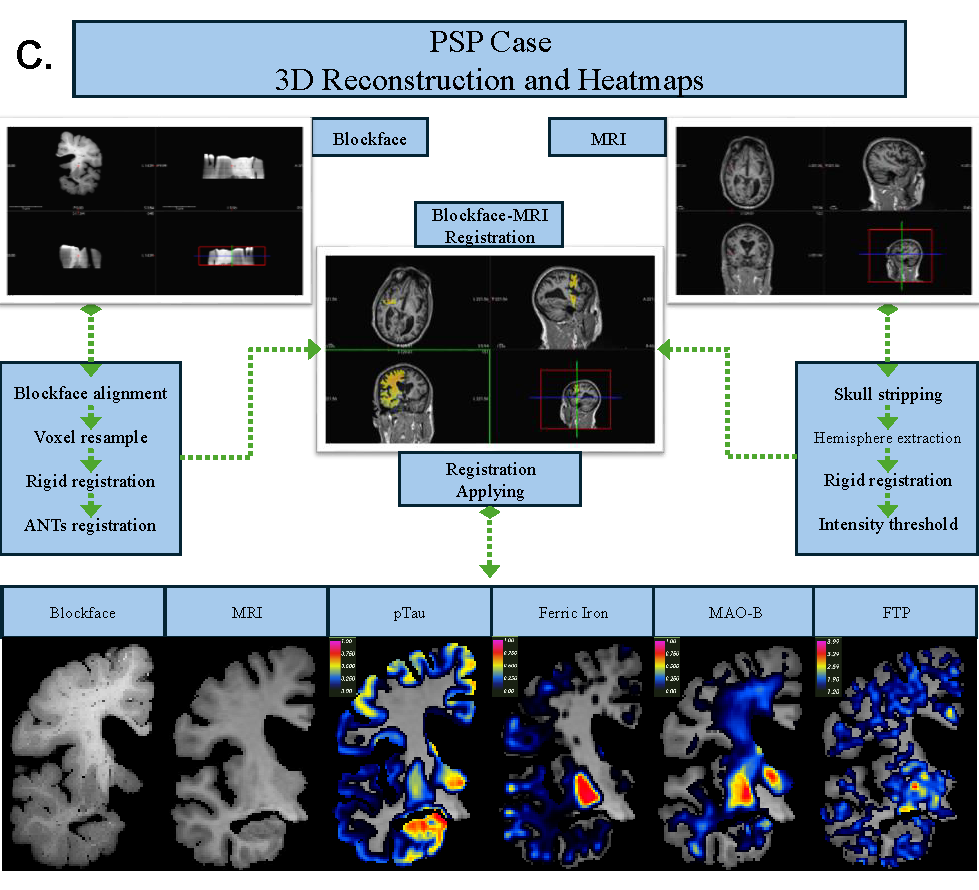
**

**
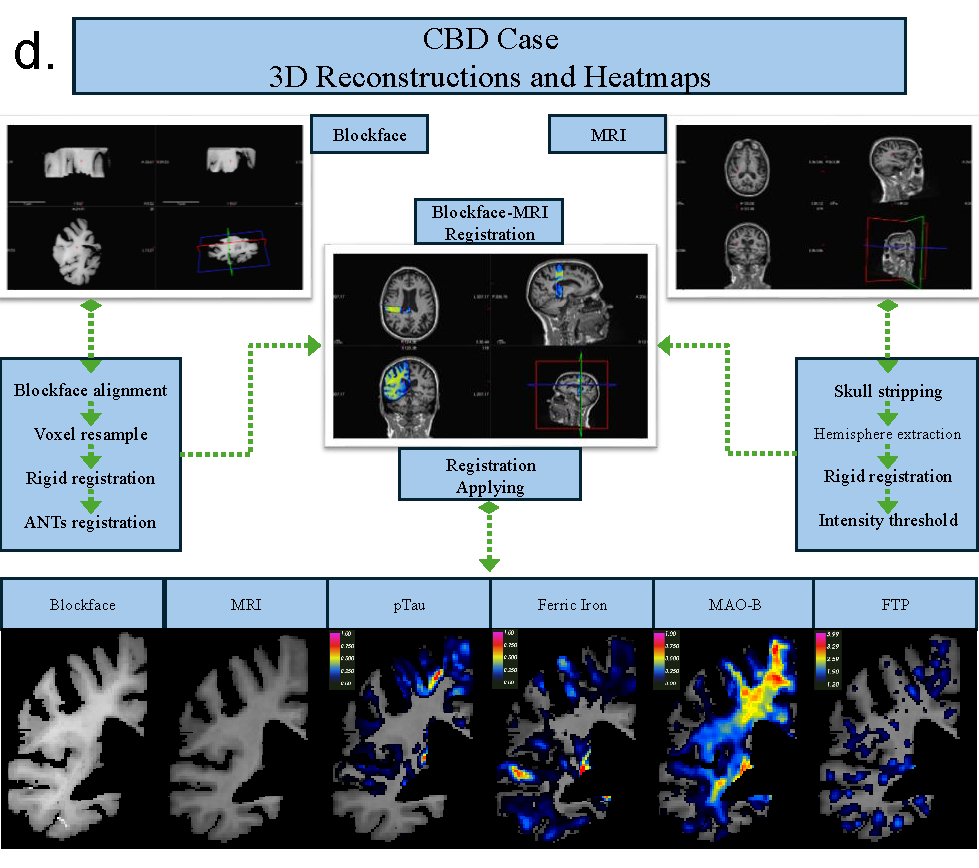
**

**
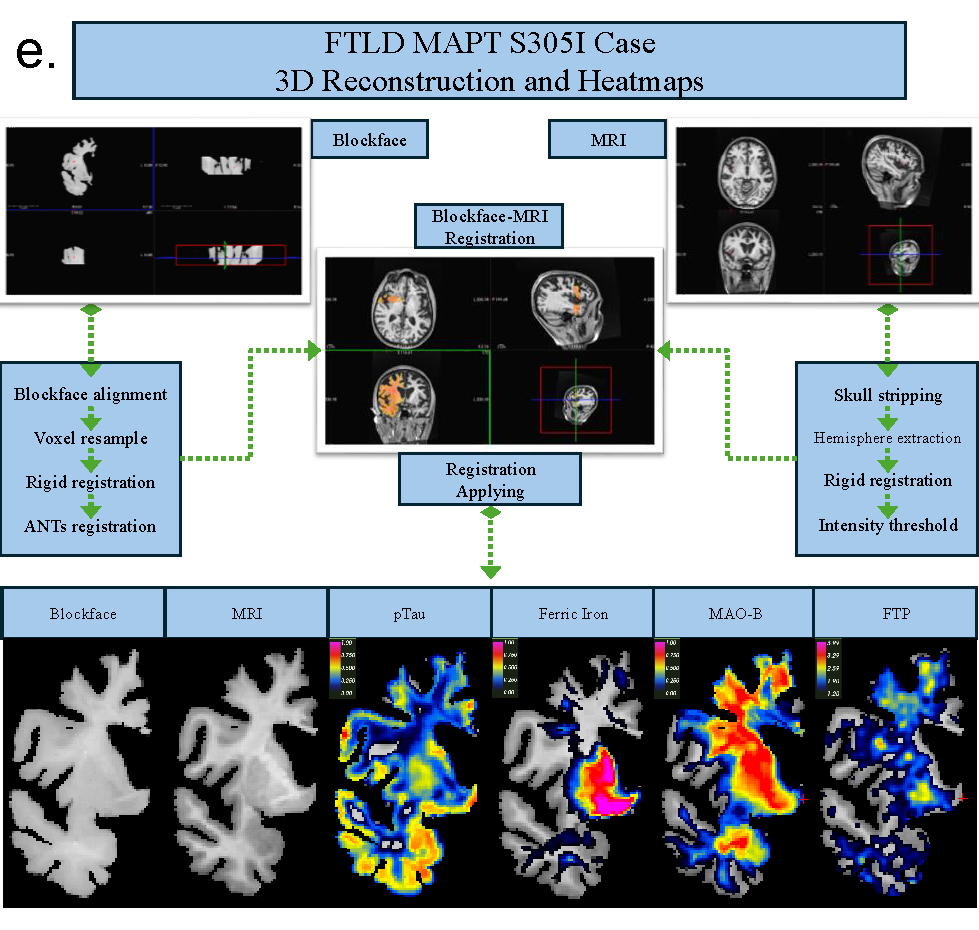
**

**
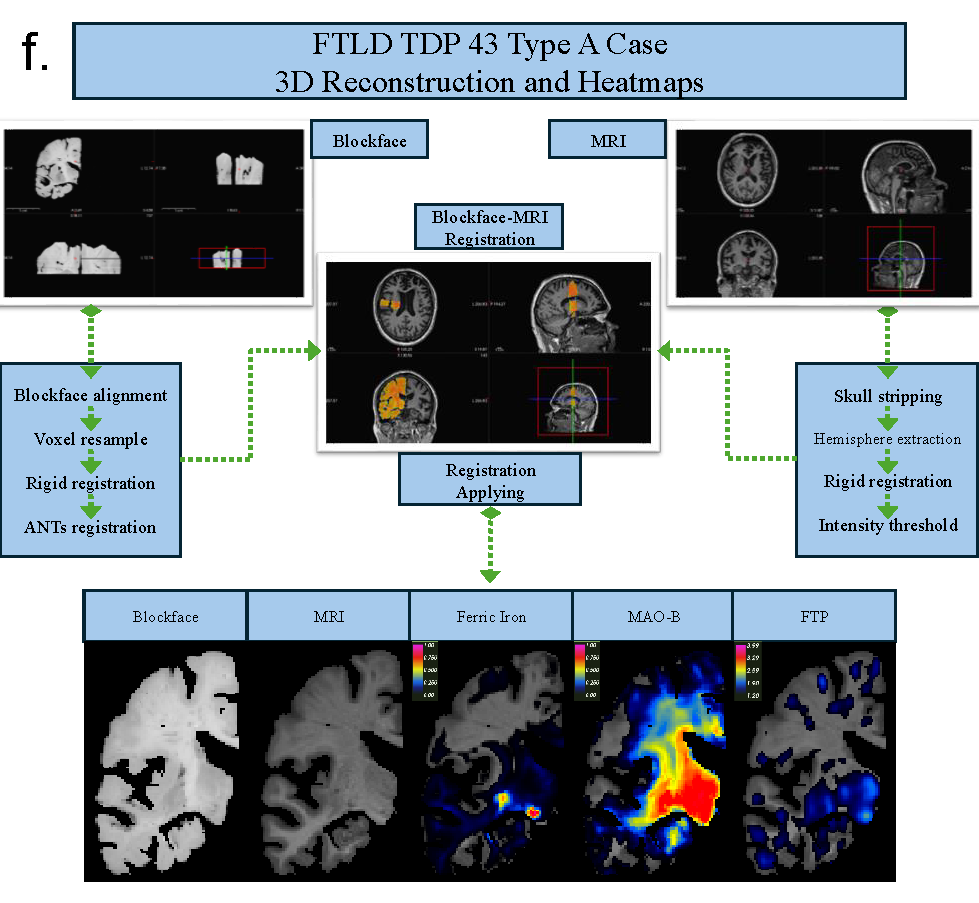
**

**Fig. S9 Dice coefficient analysis of registration accuracy between histological slabs and MRI.** Panels (a–f) show overlays of MRI-derived hemisphere masks on the corresponding histological masks used to calculate whole-slab Dice coefficients for each case. For cases in which the putamen and globus pallidus were present, Dice coefficients were also calculated for these structures (g–i). Abbreviations: AD, Alzheimer disease; CBD, corticobasal degeneration; CC, corpus callosum; MRI, magnetic resonance imaging; PH, posterior hippocampus; PSP, progressive supranuclear palsy; ROI, region of interest; FTLD, frontotemporal lobar degeneration; MAPT, microtubule-associated protein tau; TDP-43, transactive response DNA-binding protein 43.

**
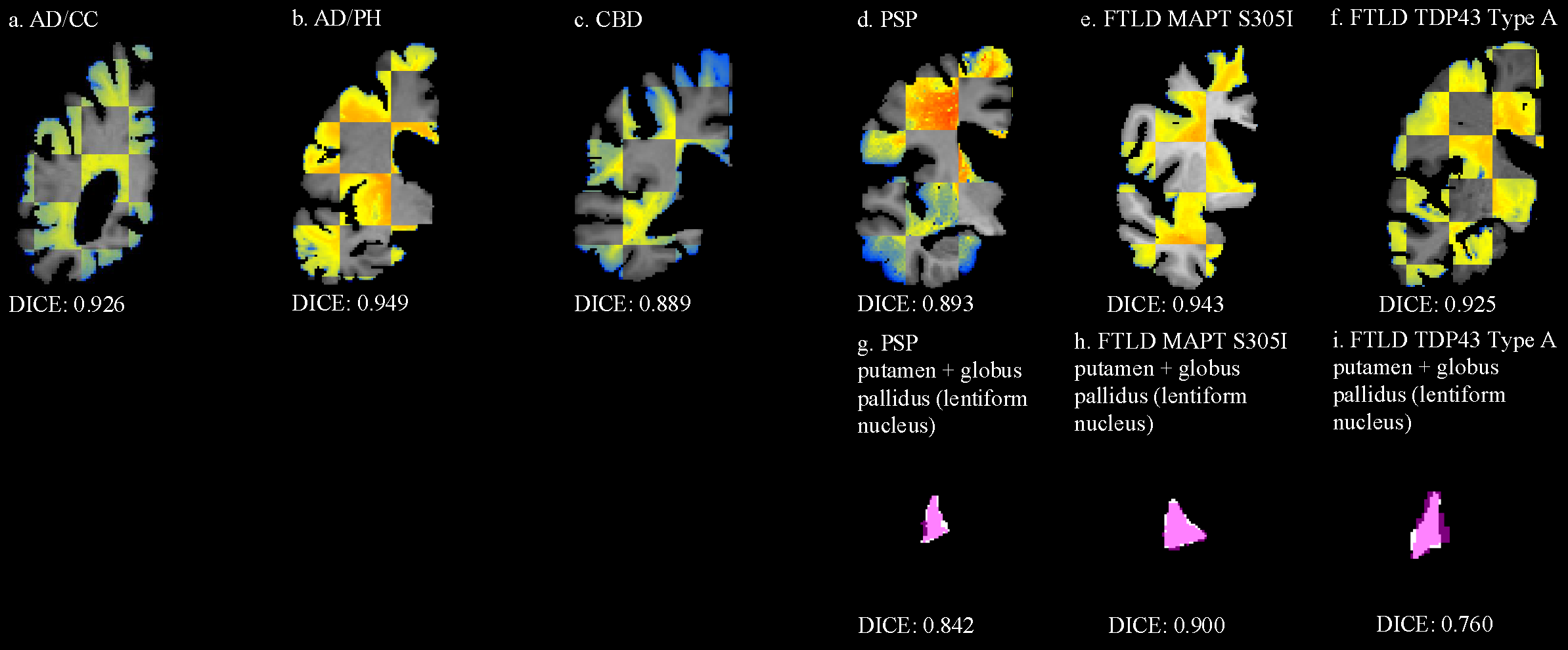
**

**RESULTS**

**Fig. S10 Voxel-wise distributions of Flortaucipir SUVR and histological signals across ROIs.** Hexagonal-binned scatter plots illustrating voxel clusters by signal intensity. Each subplot corresponds to a FreeSurfer-derived ROI mask (full hemisphere, cortex, white matter, lenticular nucleus, and hippocampus) applied across all five cases (AD, PSP, CBD, FTLD MAPT-S305I, and FTLD TDP-43 type A; AD includes combined CC and PH slabs). Each hexagonal bin represents the number of voxels within a 1/20 interval along the x- and y-axes; darker colors indicate bins containing more voxels (see individual scale bars for exact counts). The x-axis shows histological signal (p-tau/CP-13, ferric iron, or MAO-B) normalized from 0 to 1, and the y-axis shows Flortaucipir SUVR from 1.2 to 4.


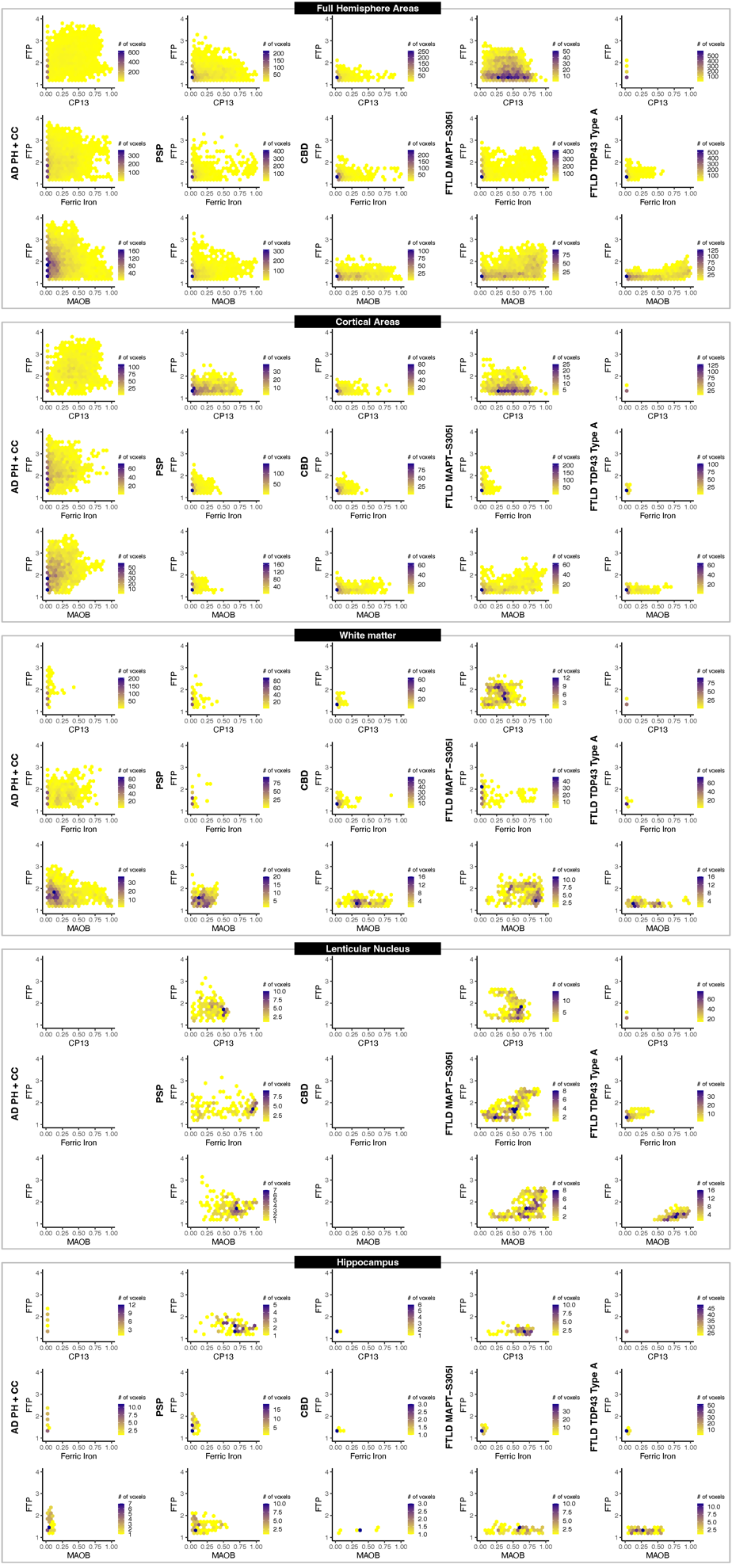


**Table S1 Comparison of Spearman Correlation Under Different Flortaucipir PET SUVR Threshold:** This statistical analysis shows how Spearman correlation between Flortaucipir PET and histological signal (p-tau/CP-13, Ferric Iron and MAO-B) across different SUVR thresholds may change for all cases in this study including AD (CC + PH combined and separately), PSP, CBD, FTLD MAPT-S305I and FTLD TDP43 Type A. Thresholds are set to be 1.2 (used in this study), 1.3 and 1.4. Each sub-table provides number of voxel after thresholding, the Spearman correlation coefficient (Rho) between the specific histology to Flortaucipir PET within that threshold, the corresponding p-value, and the difference between Rho with higher SUVR threshold (1.3 and 1.4) and Rho with 1.2 SUVR threshold.


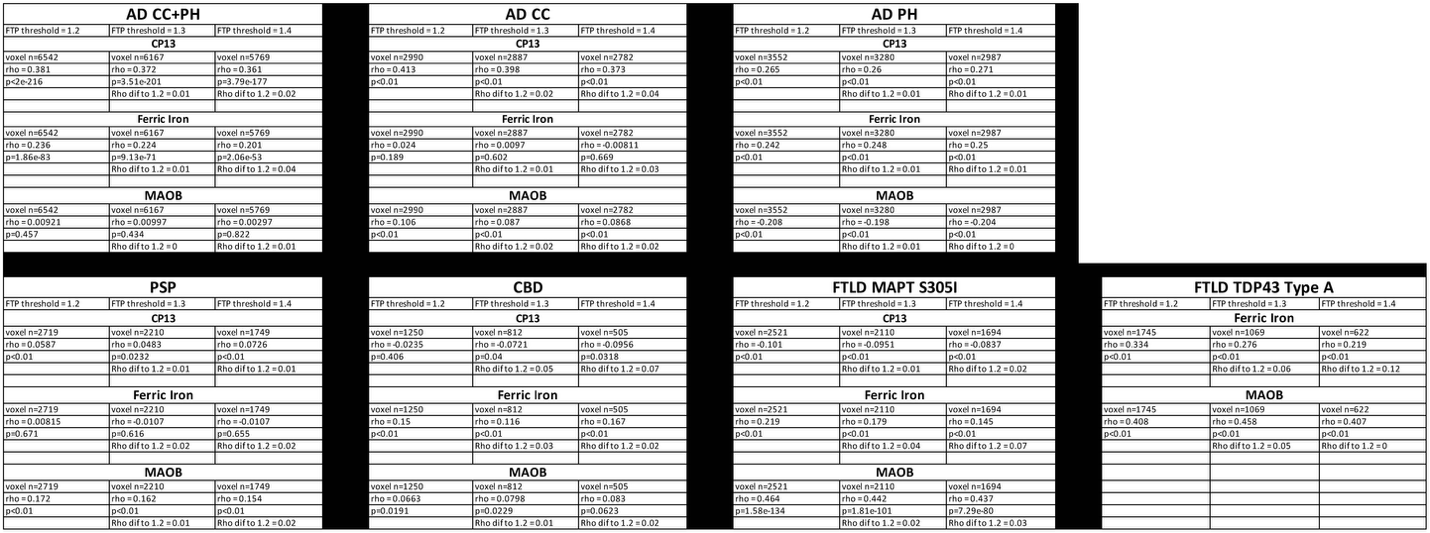


**REFERENCES**

1. Ushizima, D., et al., *Deep learning for Alzheimer's disease: Mapping large-scale histological tau protein for neuroimaging biomarker validation.* Neuroimage, 2022. **248**: p. 118790.

2. Theofilas, P., et al., *A novel approach for integrative studies on neurodegenerative diseases in human brains.* J Neurosci Methods, 2014. **226**: p. 171-83.

3. Bria, A. and G. Iannello, *TeraStitcher - a tool for fast automatic 3D-stitching of teravoxel-sized microscopy images.* BMC Bioinformatics, 2012. **13**: p. 316.

4. Schindelin, J., et al., *Fiji: an open-source platform for biological-image analysis.* Nat Methods, 2012. **9**(7): p. 676-82.

5. Fan, R.E., et al., *LIBLINEAR: A Library for Large Linear Classification*  J. Mach. Learn. Res., 2008. **9**: p. 1871–1874.

6. Ronneberger, O., P. Fischer, and T. Brox. *U-Net: Convolutional Networks for Biomedical Image Segmentation*. in *Medical Image Computing and Computer-Assisted Intervention – MICCAI 2015*. 2015. Cham: Springer International Publishing.

7. Smith, L.N., *cyclical learning Rates for Training Neural Networks.* ArXiv150601186 Cs, 2015.

8. Jianbo, S. and J. Malik, *Normalized cuts and image segmentation.* IEEE Transactions on Pattern Analysis and Machine Intelligence, 2000. **22**(8): p. 888-905.

9. Avants, B.B., et al., *The Insight ToolKit image registration framework.* Front Neuroinform, 2014. **8**: p. 44.

10. Zack, G.W., W.E. Rogers, and S.A. Latt, *Automatic measurement of sister chromatid exchange frequency.* J Histochem Cytochem, 1977. **25**(7): p. 741-53.

11. McAuliffe, M.J., et al. *Medical Image Processing, Analysis and Visualization in clinical research*. in *Proceedings 14th IEEE Symposium on Computer-Based Medical Systems. CBMS 2001*. 2001.

12. Beg, M.F., et al., *Computing Large Deformation Metric Mappings via Geodesic Flows of Diffeomorphisms.* International Journal of Computer Vision, 2005. **61**(2): p. 139-157.

13. Van Rossum, G. and F.L. Drake Jr, *Python tutorial.* . 1995, Centrum voor Wiskunde en Informatica: Amsterdam, The Netherlands.

14. Fischl, B., *FreeSurfer.* Neuroimage, 2012. **62**(2): p. 774-81.
